# Prognostic predictions in psychosis: exploring the complementary role of machine learning models

**DOI:** 10.1136/bmjment-2025-301594

**Published:** 2025-06-26

**Authors:** Violet van Dee, Seyed M Kia, Caterina Fregosi, Wilma E Swildens, Anne Alkema, Albert Batalla, Coen van den Berg, Danko Coric, Edwin van Dellen, Lotte G Dijkstra, Arthur van den Doel, Livia S Dominicus, John Enterman, Frank L Gerritse, Marte Z van der Horst, Fedor van Houwelingen, Charlotte S Koch, Lisanne E M Koomen, Marjan Kromkamp, Michelle Lancee, Brian E Mouthaan, Diane F van Rappard, Eline J Regeer, Raymond W J Salet, Metten Somers, Jorgen Straalman, Marjolein H T de Vette, Judith Voogt, Inge Winter-van Rossum, Rene S Kahn, Wiepke Cahn, Hugo G Schnack

**Affiliations:** 1 Psychiatry, University Medical Centre Utrecht Brain Centre, Utrecht, The Netherlands; 2 Psychiatry, St Antonius Hospital, Utrecht, The Netherlands; 3 Department of Cognitive Science and Artificial Intelligence, Tilburg University, Tilburg, The Netherlands; 4 Department of Informatics, System and Communication, University of Milan-Bicocca, Milan, Italy; 5 Altrecht Mental Health Care Institute, Utrecht, The Netherlands; 6 Inholland University of Applied Sciences, Amsterdam, The Netherlands; 7 University Medical Centre Utrecht Brain Centre, Utrecht, The Netherlands; 8 GGZ Delfland, Delft, The Netherlands; 9 Neurology, UZ Brussel, Brussels, Belgium; 10 Parnassia Group, The Hague, The Netherlands; 11 Psychiatry, Tergooi MC, Hilversum, The Netherlands; 12 Mediant Mental Health Care, Enschede, The Netherlands; 13 BuurtzorgT Mental Health Care, Utrecht, The Netherlands; 14 Psychiatry, Icahn School of Medicine at Mount Sinai, New York, New York, USA; 15 Institute for Language Sciences, Utrecht University, Utrecht, The Netherlands

**Keywords:** Schizophrenia & psychotic disorders, Machine Learning

## Abstract

**Background:**

Predicting outcomes in schizophrenia spectrum disorders is challenging due to the variability of individual trajectories. While machine learning (ML) shows promise in outcome prediction, it has not yet been integrated into clinical practice. Understanding how ML models (MLMs) can complement psychiatrists’ predictions and bridge the gap between MLM capabilities and practical use is key.

**Objective:**

This vignette study aims to compare the performance of psychiatrists and MLMs in predicting short-term symptomatic and functional remission in patients with first-episode psychosis and explore whether MLMs can improve psychiatrists’ prognostic accuracy.

**Method:**

24 psychiatrists predicted symptomatic and functional remission probabilities at 10 weeks based on written baseline information from 66 patients in the OPtimization of Treatment and Management of Schizophrenia in Europe (OPTiMiSE) trial. ML-generated predictions based on these vignettes were then shared with psychiatrists, allowing them to adjust their estimates.

**Findings:**

The predictive accuracy of the MLM was low but comparable to that of psychiatrists for symptomatic remission (MLM: 0.50, psychiatrists: 0.52) and comparable to that of psychiatrists for functional remission (MLM: 0.72, psychiatrists: 0.79). Inter-rater agreement was low but comparable for psychiatrists and the MLM. Although the MLM did not improve overall predictive accuracy, it showed potential in aiding psychiatrists with difficult-to-predict cases. However, psychiatrists struggled to recognise when to rely on the model’s output, and we were unable to determine a clear pattern in these cases based on their characteristics.

**Conclusions:**

MLMs may have the potential to support psychiatric decision-making, particularly in difficult-to-predict cases, but at present, their effectiveness remains limited due to constraints in predictive accuracy and the ability to identify when to rely on the model’s output. Addressing these issues is crucial to improve the utility of MLMs and foster their integration into clinical practice.

**Clinical implications:**

MLMs are best suited as supplementary tools, providing a second opinion while psychiatrists retain decision-making autonomy. Integrating predictions from both sources may help reduce individual biases and improve accuracy. This approach leverages the strengths of MLMs without compromising clinical responsibility.

**WHAT IS ALREADY KNOWN ON THIS TOPIC**

- While machine learning models (MLMs) show promise in predicting outcomes in psychotic disorders, they have yet to be integrated into clinical practice. Evidence on the predictive accuracy of psychiatrists for these disorders is limited, with only two small studies published before 1990 suggesting moderate accuracy. Comparisons of MLMs and psychiatrists in this context have not been previously conducted.

**WHAT THIS STUDY ADDS**

- This is the first study to compare the predictive accuracy of psychiatrists with that of an MLM for psychotic disorders and to assess whether an MLM can enhance psychiatrists’ performance. It highlights that while MLMs do not improve overall accuracy, they may support psychiatrists in difficult cases.

**HOW THIS STUDY MIGHT AFFECT RESEARCH, PRACTICE OR POLICY**

- The findings emphasise the need for advancements in MLM accuracy, interpretability and strategies to identify cases where MLMs are most beneficial. These improvements could foster effective integration of MLMs as supplementary tools in clinical practice, aiding psychiatrists in decision-making while maintaining their autonomy.

## Background

 Schizophrenia spectrum disorders (SSDs) have highly variable outcomes, making accurate and individualised outcome prediction — essential to personalised care — a challenging task. The reported rates of remission and recovery in first-episode schizophrenia vary considerably across studies, depending on factors such as treatment setting, duration of follow-up and definition of outcome. A recent systematic review and meta-analysis (mean follow-up 44 months) reported that approximately 54% of patients achieve symptomatic remission and 32% achieve functional remission.^1^ However, few studies have assessed prognosis within the first 12 weeks of treatment. Although full recovery is often only achieved later in the course of illness, early treatment response is a key clinical indicator. It can inform decisions about the intensity and duration of care, guide patient and family expectations and potentially serve as a predictor of long-term outcomes.^2^ One study reported a 71% symptomatic remission rate at 12 weeks in patients with first-episode non-affective psychosis.^3^ To our knowledge, no studies have examined functional remission rates within this short time frame.

In psychosis prognosis prediction, machine learning models (MLMs) are trained to identify complex, often non-linear relationships between baseline patient data and future clinical outcomes. By analysing large data sets containing features such as demographics, clinical symptoms, cognitive test scores and neuroimaging data, these models aim to predict outcomes like treatment response, functional recovery or symptom persistence. MLMs can uncover subtle patterns that may not be evident to clinicians, thereby enabling more personalised prognostic insights for individuals with early psychosis.

The integration of MLMs into clinical psychiatry presents a promising opportunity to enhance data-driven decision-making in the prediction of psychosis outcomes. Over the past two decades, numerous studies have explored the use of MLMs for outcome prediction in SSD.^4 5^ However, many of these studies are limited by small sample sizes and a lack of external validation, weakening the robustness of their findings.^4 6^

Despite these limitations, ongoing advances in algorithmic predictive power signal that the time has come to bridge the gap between MLM capabilities and clinical practice. Currently, no MLMs for psychiatric disorders, including SSDs, have been integrated into clinical practice yet.^7^ Establishing their clinical value requires comparison to clinicians’ predictions, particularly regarding accuracy, inter-rater reliability and potential to assist or augment traditional assessments.

Current studies mainly assess MLMs by comparing their predictive accuracy to random chance. For clinical relevance, however, MLM performance should be compared with clinicians’ accuracy. If an MLM performs at least as well as the psychiatrist, it could potentially assist or replace the psychiatrist for this task. Little data exists on the accuracy and inter-rater reliability of psychiatrists’ outcome predictions.^8^

Psychiatrists’ predictions rely on demographic and clinical information, Diagnostic and Statistical Manual of Mental Disorders, Fifth Edition (DSM 5) classifications, personal clinical experience and ‘skilled’ intuition.^9 10^ The multifactorial aetiology of psychiatric symptoms, the lack of objective biomarkers, incomplete information, questionable validity of psychiatric classifications and susceptibility of clinical impression to bias may contribute to low accuracy and low inter-rater agreement of psychiatrists in clinical predictions.^9 11^ Regarding SSDs, only two small studies published before 1990 have investigated the prognostic accuracy of outcome prediction by clinicians.^12 13^ In the first study, psychiatrists’ predictions on both clinical and functional outcome parameters after 1 year scarcely outperformed chance statements.^12^ The second study reported ‘acceptable agreement’ between predictions and true outcome, particularly for clinical outcomes (‘length of psychotic episode’ and ‘time spent in hospital’) compared with functional outcomes (‘occupational capacity’ and ‘functioning in family’).^13^ Discrepancies in predictive accuracy between studies might stem from differences in defining the criteria for correct predictions between studies. Inter-rater reliability of psychiatrists in SSD remains unexamined.

### Objective

In our study, psychiatrists and MLM predicted individual chance of symptomatic and functional remission at 10 weeks for 66 patients with first episode psychosis, solely based on the collected baseline information. With the generated predictions, we compared the predictive performance of psychiatrists with that of the MLM and explored whether the MLM helped psychiatrists to enhance the accuracy of their prognosis predictions.

## Methods

### Study design

This vignette study investigates the predictive accuracy of psychiatrists and MLM in predicting symptomatic and functional remission in people with first episode psychosis. This study was preregistered on AsPredicted.org (protocol #132306).

### Data collection

#### OPTiMiSE data set

In this study, data from the OPtimization of Treatment and Management of Schizophrenia in Europe (OPTiMiSE) trial were used (trial identifier number NCT01248195).^14^ The primary objective of the OPTiMiSE trial was to establish a treatment algorithm for individuals with first episode schizophrenia. In this OPTIMISE trial ‘first episode of schizophrenia’ is defined as a DSM-IV diagnosis of schizophrenia, schizophreniform disorder or schizoaffective disorder in individuals aged 18–40, with psychosis onset within the last 2 years. Additionally, the person must have used antipsychotic medication for no more than 2 weeks in the past year or 6 weeks over their lifetime.

The first phase involved 446 patients undergoing amisulpride treatment for 4 weeks. This phase was completed by 371 patients. Patients meeting the symptom severity component of the consensus criteria for symptomatic remission of the Remission in Schizophrenia Working Group (RSWG) automatically completed the study.^15^ Subsequently, the 93 patients not in remission proceeded to the second phase, where they were randomly assigned to either continue amisulpride or switch to olanzapine for an additional 6 weeks in a double-blind fashion.

For the current study, data from the 66 patients that completed the second phase with complete Positive And Negative Symptom Scale (PANSS) and Personal and Social Performance Scale (PSP) records were used. Characteristics of these patients are available in online supplemental file 1. For all included patients the ‘true outcome’ at 10 weeks follow-up was established. Symptomatic remission was defined according to the symptom severity component of the RSWG criteria.^15^ Functional remission was defined as a score of 71 points and higher on the PSP, because a score between 71 and 100 refers to only mild difficulties.^16^

#### Machine learning model

The psychosis prognosis prediction model developed by van Opstal *et al*^17^ was employed in this study. This model, based on a recurrent neural network architecture, integrates multimodal data from diverse sources and can simultaneously predict multiple outcome measures. For this study, we specifically applied the S3 scenario developed by van Opstal *et al*. (2024), in which baseline data (week 0) were used to predict symptomatic and functional outcomes at week 10. The model was trained using available historical data from all patients in the OPTiMiSE trial at baseline, excluding those allocated to the test set. Baseline patient information included demographic, lifestyle, somatic and diagnostic data, as well as responses from multiple symptom screening and scoring questionnaires (table 1). The model received 229 input features across various modalities. The benchmarked data consisted solely of the 72 patients who completed the second phase (week 10) of the OPTiMiSE trial. To address potential overfitting, the model was first pretrained on 10 000 synthetically generated samples drawn from realistic feature distributions. Additionally, data augmentation was applied using a variable-length sliding window approach. Regularisation techniques included dropout throughout the model and Monte Carlo dropout at inference time to estimate uncertainty. Due to instability concerns with small samples, feature selection was not applied (see van Opstal *et al* (2024), online supplemental information). Instead, the model architecture was designed to be modular and regularised to enhance generalisation.

**Table 1:** Patient information and measurements

Type	Number of features	Features
Baseline information available to psychiatrists and MLM for making predictions		
Demographic	20	Age (con), sex (bin), race (cat), immigration status (bin), marital status (bin), divorce status (bin), occupation status (bin), occupation type (cat), previous occupation status (bin), previous occupation type (cat), father’s occupation (cat), mother’s occupation (cat), years of education (con), highest education level (cat), father’s highest degree (cat), mother’s highest degree (cat), living status (bin), dwelling (cat), income source (cat), living environment (cat)
Diagnostic	7	DSM-IV classification (cat), duration of the current psychotic episode (con), current psychiatric treatment (cat), psychosocial interventions status (bin), estimated prognosis (cat), hospitalisation status (bin)
Lifestyle	7	Recreational drugs history (bin), recreational drugs since last visit (bin), caffeine drinks per day (con), last caffeine drink (cat), drink alcohol (bin), alcoholic drinks in the last year (cat), smoking status (bin)
Somatic	11	Height (con), weight (con), waist (con), hip (con), BMI (con), systolic blood pressure (con), diastolic blood pressure (con), pulse (con), ECG abnormality (bin), last mealtime (cat), last meal type (cat)
Treatment	1	Average medication dosage (con)
CDSS	9	Calgary Depression Scale for schizophrenia (con)
SWN-K	20	Subjective Well-being under Neuroleptic Treatment Scale (con)
MINI*	48	Mini International Neuropsychiatric Interview
PANSS	30	Positive And Negative Symptom Scale (con)
PSP	5	Personal and Social Performance Scale (con)
CGI	2	Clinical Global Impression scale severity and improvement (con)
10 weeks follow-up information used for determining the true remission status		
PANSS	8	Positive And Negative Symptom Scale items: P1–3, N1, N4, N6, G5, G9 (con)
PSP	5	Personal and Social Performance Scale (con)

* The MINI is a structured psychiatric diagnostic assessment covering 48 psychiatric (co)morbidities in both the present and past.

bin, binary measure; cat, categorical measure; con, continuous measure; DSM-IV, Diagnostic and Statistical Manual of Mental Disorders, Fourth Edition; MLM, machine learning model.

#### Participants

12 psychiatrists and 12 residents in psychiatry, each with at least 1 year of clinical experience with severe mental illness, participated in the study. In the remainder of this paper, for the purpose of clarity and conciseness, all participants will be referred to as psychiatrists, unless stated otherwise. Written informed consent was obtained from all participants.

A pilot study showed that reaching a prognosis based on patient information was time-consuming for psychiatrists. To prevent participants from dropping out due to time constraints or the accuracy of predictions being affected by fatigue, we decided to divide the psychiatrists into three groups. Each participant group consisted of four psychiatrists and four residents in psychiatry. We aimed to distribute the level of experience in working with patients with psychotic disorders equally among the groups. The cases were also divided into three groups (group 1–3), with each group of psychiatrists being assigned one set of 22 cases.

#### Instruments

All participants completed a questionnaire (online supplemental file 2) in the Castor Electronic Data Capture online secure survey software programme.^18^ All participants’ confidentiality agreements regarding the presented (pseudo-anonymised) patient data were obtained before participants commenced the real survey.

In the first part of the survey, general information about the participants (age, sex, country of birth, occupation (psychiatrist/resident in psychiatry) and years of experience working with patients with psychotic disorders) was collected.

For the second part of the survey, each participant group received baseline information on 22 (of the 66) randomly assigned patient cases from the OPTiMiSE data set (table 1, online supplemental file 1). For each case, participants predicted the chance of symptomatic and functional remission at 10 weeks on a scale of 0–100%. This first prediction is referred to as pre-MLM. Participants were also queried on the importance of specific patient information for their predictions.

Subsequently, the MLM prognosis and its level of certainty (uncertain, certain, definite) about that prognosis for the same cases,^17^ based on identical information, was presented to the participants, allowing them to adjust their predictions and provide reasoning. Whether adjusted or not, these second psychiatrists’ predictions are referred to as post-MLM.

The third part of the survey explored participants’ trust in artificial intelligence for prognosis prediction (measured on a 5-point scale), the extent to which they were inclined to consider the MLMs’ prediction (open-ended question) and whether they perceived any crucial information gaps in the patient data (open-ended question). Finally, psychiatrists were asked to estimate their predictive accuracy in this research on a scale between 0% and 100%.

### Data analysis

#### Who has better predictive performances; psychiatrists or MLM?

##### Can data from psychiatrists and residents be pooled?

In a preanalysis, we assessed whether the data of psychiatrists and residents in psychiatry could be pooled for further analysis. The mean accuracies between the groups were compared using a pooled variance t-test (Student’s t-test).

##### How accurate are the prognosis predictions of psychiatrists and MLM?

For each rater (participants and MLM), by comparing their predictions with the true outcomes, the following performance metrics were calculated: area under the receiver operating characteristic curve (AUC), accuracy, sensitivity, specificity, balanced accuracy and Brier score. Mean prognostic performances (across cases) of participants and MLM were compared using the non-parametric Mann-Whitney U tests, because the participants’ data was not distributed normally for all prognostic metrics.

##### How comparable are the prognosis predictions among participants and between participants and the MLM?

Predictive agreements at group level (all participants+MLM) and pairwise inter-rater agreements were calculated with intraclass correlation coefficients (ICCs), based on a single-rating, absolute-agreement, two-way random-effects model. In addition, the pairwise predictive agreement between participants and MLM was compared with the pairwise predictive agreement among participants by a Mann-Whitney U test.

To visualise the relationships and distribution of accuracy of prognostic predictions of participants and MLM (for participants both pre-MLM and post-MLM), we calculated the Euclidean distances between the vectors of predictions for each group. Then we performed classical multidimensional scaling (MDS) to construct rater representations in a two-dimensional space, the coordinates of which were used for locating each rater in a scatter plot.

### Can the MLM help participants to enhance the accuracy of their prognosis predictions?

#### Does the MLM help participants to enhance the accuracy of their predictions in general?

With a one-sample t-test, we calculated whether the mean difference between the accuracy of psychiatrists pre-MLM and post-MLM significantly differed from 0.

#### Does the MLM help participants to enhance the accuracy of their predictions in cases with specific characteristics?

To effectively use the MLM, as a post hoc analysis, we aimed to identify cases where the psychiatrist’s prognosis predictions were poor, but the MLM performed well. All cases were grouped based on the number of correct predictions made by psychiatrists, creating nine difficulty groups ranging from 0 to 8 correct predictions. For each group, we calculated the percentage of correct predictions (accuracy) for both the psychiatrists and the MLM. To compare the accuracy of the MLM and psychiatrists across groups, the results were visualised along with the number of cases in each group.

We then focused on the groups where psychiatrists made ≤3 correct predictions, defined as the ‘hard cases’. To statistically evaluate whether the MLM provided added value over psychiatrists in the ‘hard cases’, the difference in accuracy between the MLM and psychiatrists (MLM accuracy—psychiatrist accuracy) was calculated for each of these groups. A one-sided Wilcoxon signed-rank test was performed to test the null hypothesis that the median difference was equal to zero, that is, a non-significant advantage of the MLM in these difficult cases.

Then, we investigated whether cases in these ‘hard case’ groups shared similar characteristics that could potentially be recognised by psychiatrists (or by computers). To do this, we created a t-distributed Stochastic Neighbor Embedding (t-SNE) plot to visualise the similarity of case characteristics. Data points representing ‘hard cases’ were highlighted with a distinct colour to assess whether these cases shared more similar characteristics than others. More information about the characteristics and data preparation used in this analysis is provided in online supplemental file 8.

### Findings

The mean age of the participants was 37.8 years (SD 9.1 years) and 50% were male. The mean number of years of working experience as a medical doctor in psychiatry was 15.1 (SD 8.5) years for psychiatrists and 4.0 (SD 1.4) years for residents.

#### Predictive performances of psychiatrists and MLM

##### Prognostic accuracy of psychiatrists and residents

A pooled variance t-test (Student’s t-test) showed no significant difference in mean accuracy between the resident and psychiatrist groups (symptomatic remission t(22) = −1.15, p=0.26, and functional remission (t(22) = 0.18, p=0.86)). Based on these results, the data from residents and psychiatrists were pooled under the term ‘psychiatrists’ for further analyses.

##### Comparison of predictive performances of psychiatrists versus MLM

Predictive performances of psychiatrists and the MLM are displayed in table 2 and online supplemental file 3.

**Table 2:** Predictive performances of psychiatrists and the MLM

	Psychiatrists		MLM
	pre-MLM, mean (SD)	post-MLM, mean (SD)	
Symptomatic remission			
AUC	0.58 (0.12)	0.58 (0.10)	0.59
Accuracy	0.52 (0.13)	0.51 (0.13)	0.50
Sensitivity	0.65 (0.22)	0.71 (0.21)	0.83
Specificity	0.44 (0.26)	0.38 (0.27)	0.24
Balanced accuracy	0.54 (0.11)	0.55 (0.10)	0.54
Brier score	0.28 (0.05)	0.28 (0.05)	0.28
Functional remission			
AUC			
Without group 1*	0.76 (0.14)	0.77 (0.13)	0.77
All participants	0.70 (0.22)	0.64 (0.26)	0.67
Accuracy			
Without group 1*	0.75 (0.10)	0.75 (0.11)	0.80
All participants	0.72 (0.15)	0.72 (0.15)	0.79
Sensitivity			
Without group 1*	0.52 (0.27)	0.55 (0.27)	0.57
All participants	0.55 (0.36)	0.41 (0.36)	0.50
Specificity			
Without group 1	0.79 (0.13)	0.79 (0.13)	0.84
All participants	0.75 (0.18)	0.76 (0.18)	0.83
Balanced accuracy*			
Without group 1	0.65 (0.12)	0.67 (0.13)	0.70
All participants	0.65 (0.16)	0.58 (0.17)	0.66
Brier score			
Without group 1	0.16 (0.05)	0.16 (0.04)	0.17
All participants	0.17 (0.05)	0.17 (0.05)	0.17

* Because ‘functional remission’ occurred in only 1/22 of the randomly assigned cases in group 1, results of the sensitivity and therefore also AUC and balanced accuracy are not reliable/representative.

AUC, area under the receiver operating characteristic curve; MLM, machine learning model.

The mean prognostic performances — AUC, accuracy, sensitivity, specificity, balanced accuracy and Brier score — of psychiatrists and MLM, for both symptomatic and functional remission, showed no significant differences (all p values>0.5).

##### Comparison of predictive agreement among psychiatrists and between psychiatrists and MLM

Predictive agreement (ICC) of all raters (psychiatrist and MLM) at group level was in general poor with all group ICCs <0.5 for both symptomatic and functional remission (table 3, online supplemental file 4 — Pairwise ICC matrices).^19^ For both symptomatic and functional remission, the pairwise psychiatrist–MLM ICCs were significantly lower than psychiatrist–psychiatrist ICCs for group 3 (symptomatic remission p=0.02, functional remission p=0.001), but not for groups 1 and 2.

**Table 3:** Predictive agreement by intraclass correlation coefficients (ICC)*

	Overall ICC (95% CI)	Lowestpairwise ICC	Highestpairwise ICC	Pairwise ICCs >0.5 (n)	Comparison of ICC distribution psychiatrist–MLM versus psychiatrist–psychiatrist by Mann-Whitney U-test
Symptomatic remission					
Group 1					
PSY only	0.08 (0.01 to 0.22)	−0.16	0.48	0/36	
PSY+MLM	0.07 (0.01 to 0.20)	−0.47	0.48	0/36	p=0.99
Group 2					
PSY only	0.34 (0.19 to 0.55)	0.02	0.76	3/36	
PSY+MLM	0.33 (0.18 to 0.53)	−0.13	0.76	3/36	p=0.49
Group 3					
PSY only	0.35 (0.20 to 0.56)	−0.04	0.66	8/36	
PSY+MLM	0.31 (0.17 to 0.51)	−0.04	0.66	8/36	p=0.02
Functional remission					
Group 1					
PSY only	0.22 (0.10 to 0.41)	−0.07	0.54	1/36	
PSY+MLM	0.20 (0.09 to 0.38)	−0.11	0.54	1/36	p=0.27
Group 2					
PSY only	0.39 (0.23 to 0.59)	0.06	0.77	8/36	
PSY+MLM	0.37 (0.22 to 0.57)	−0.1	0.77	9/36	p=0.32
Group 3					
PSY only	0.42 (0.26 to 0.62)	0.14	0.71	5/36	
PSY+MLM	0.36 (0.22 to 0.57)	0.03	0.71	5/36	p<0.01

* The 66 cases from the OPTiMiSE trial were randomly assigned to three groups, each making predictions for 22 unique patient cases. As inter-rater agreement may be influenced by specific case characteristics, results are presented separately for each group.

MLM, machine learning model; OPTiMiSE, OPtimization of Treatment and Management of Schizophrenia in Europe; PSY, psychiatrists.

MDS of the relationships and distributions of predictions on case-level for psychiatrists and MLM showed that the MLM’s predictions were largely similar to those of psychiatrists. In all plots, the MLM’s data point was positioned at the outer edge of the cloud of data points. However, in most plots, there were psychiatrists who deviated from the general cloud as well. Psychiatrists’ predictions post-MLM were more similar (closer) to the MLM than those pre-MLM (online supplemental file 5).

### Influence of the MLM on prognostic accuracy of psychiatrists

Predictive performances of psychiatrists pre-MLM and post-MLM are displayed in table 2. About 25% of all predictions of psychiatrists were changed post-MLM. Some psychiatrists made no changes while others made changes in up to 77% of their predictions. The amount of per cent change per prediction was highly variable as well, with the mean absolute amount of change per prediction ranging from 0% to 47%.

Approximately the same amount of predictions was changed in the correct direction as in the incorrect direction post-MLM, both for symptomatic and functional remission (online supplemental files 6 and 7).

#### Influence of the MLM on mean prognostic accuracy of psychiatrists

The mean difference of accuracies of psychiatrists pre-MLM and post-MLM did not differ significantly from 0 for both symptomatic (t(23)= −0.84, p=0.41) and functional remission (t(23)= −3.61e-16, p=1) predictions.

#### Potential value of the MLM in cases that are difficult to predict for psychiatrists

The post hoc analysis of case categorisation based on the number of psychiatrists that predicted them correctly showed that for functional remission, many cases were easy to predict by the psychiatrists (nearly 50% of cases correct by ≥7 psychiatrists), while predicting symptomatic remission appeared more difficult for them (18% of cases correct by ≥7 psychiatrists).

**Figure 1:**
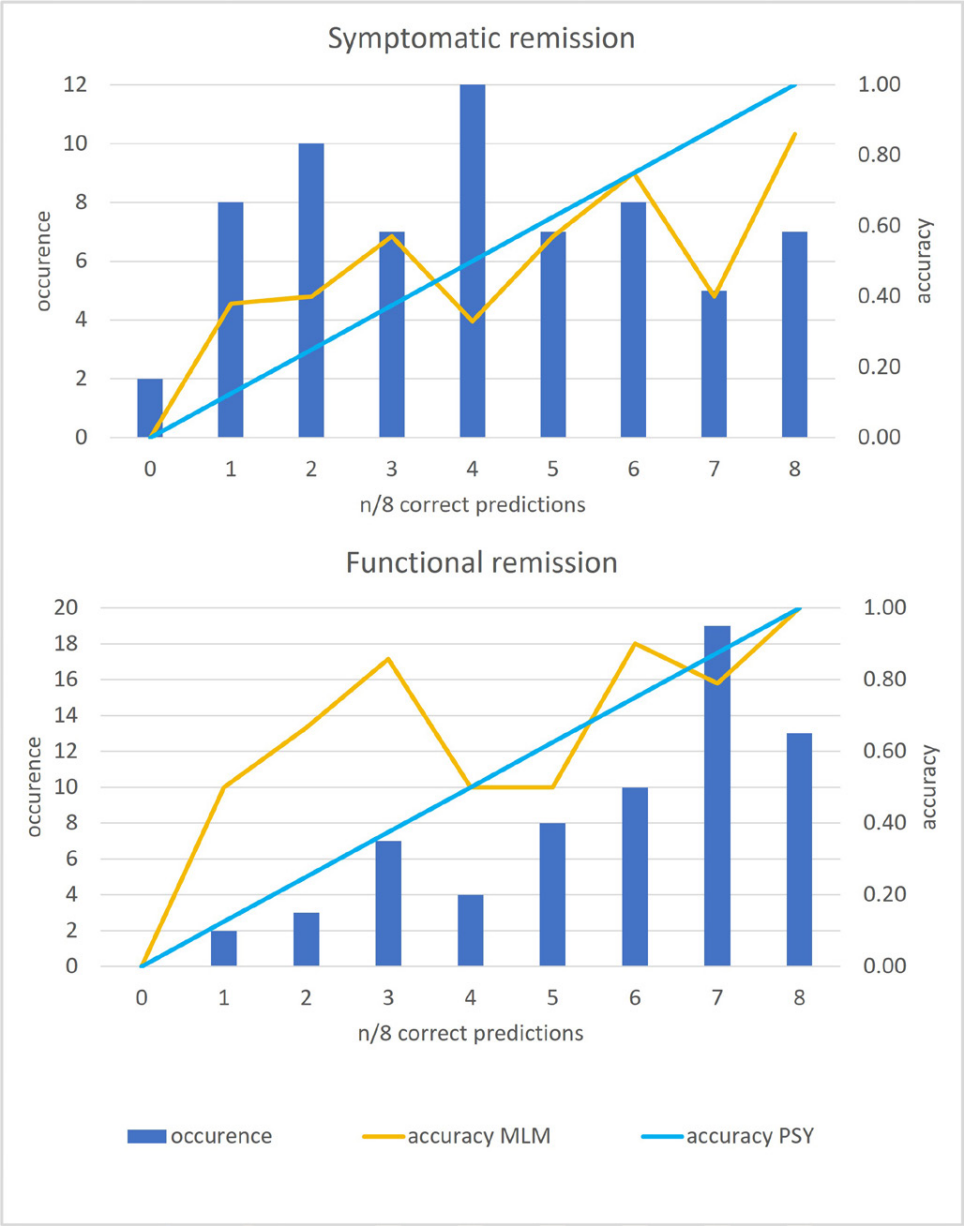
The 66 cases are grouped based on the number of psychiatrists who correctly predicted them. The blue bars represent the number of cases in each group (‘occurrence’), while the blue line indicates psychiatrist accuracy (number of correct predictions divided by 8), and the orange line shows MLM accuracy. For both symptomatic and functional remission, MLM accuracy is higher than psychiatrist accuracy in the ‘hard cases’ (≤3 correct predictions by psychiatrists). The difference in accuracy was (just) not statistically significant, with a p value of 0.06 for both symptomatic and functional remission. MLM, machine learning model; PSY, psychiatrists.

For symptomatic remission, the ‘hard case’ groups (correct by ≤3 psychiatrists, 41% of cases) appeared to be more difficult to predict by the MLM as well (figure 1). For functional remission, for the ‘hard cases’ groups (18% of cases), the accuracy of the MLM remained 50% or higher (figure 1). In the ‘hard case’ groups, the MLM demonstrated higher accuracy than the psychiatrists in each group (figure 1). However, the difference in accuracy was (just) not significant, with a p value of 0.06 for both symptomatic and functional remission.

The t-SNE plots showed that the case characteristics of the ‘hard cases’ were not distinct from the other cases (ie, no outliers), making it impossible to identify the hard cases based on case characteristics (online supplemental file 8).

## Discussion

### Summary and interpretation of key findings

Accurate, personalised short-term outcome prediction is essential for tailoring care to individual patients. This study explored whether MLMs could improve prognosis prediction in first-episode psychosis using data from the OPTiMiSE study. While predictive accuracy for symptomatic remission was only slightly above chance level and functional remission remained low, we found that the mean predictive performance of the MLM was similar to that of psychiatrists for both symptomatic and functional remission. Inter-rater agreement among psychiatrists and between psychiatrists and the MLM was low but similar across groups.

Approximately 25% of psychiatrists’ predictions were altered after exposure to the MLM’s output. The interindividual variation in both the frequency and magnitude of these changes was substantial, with roughly equal proportions of corrections in the right and wrong direction. The MLM did not help to improve predictive accuracy of psychiatrists in general. However, MDS of the predictions revealed that although the MLM’s outputs were largely similar to those of psychiatrists, they often exhibited subtle deviations, as indicated by its position at the outer edge of the cloud. This slightly different predictive approach may help explain why the MLM occasionally outperformed psychiatrists in hard cases. A critical issue was psychiatrists’ difficulty in recognising when to rely on the model, and we could not identify a clear pattern to identify ‘hard cases’ based on their characteristics.

Surprisingly, we found no difference in accuracy between psychiatrists and psychiatry residents, which contrasts with previous studies suggesting that clinical experience improves decision-making accuracy.^20^ One explanation for this discrepancy could be the absence of direct patient interaction in this study, which prevented participants from forming a ‘clinical impression’. The clinical impression might get better with increasing experience, improving predictive accuracy in a real-world setting.

### Limitations

First, the prediction accuracy in this study may have been influenced by limitations in the available patient information. Psychiatrists and MLM relied solely on case data without (information on) direct patient interaction, and the OPTiMiSE data set lacked key clinical predictors such as family history of SSD,^21^ trauma history,^22^ motivation and adherence to treatment^23 24^ and premorbid functioning.^25^

Second, the small sample size and low inter-rater agreement may have limited the detection of small effects. Due to the random assignment, group 1 contained only one patient who achieved functional remission, affecting the reliability of calculations for this group.

Third, the 10-week follow-up may have been too short to achieve functional remission, which typically takes more time.^26^

Fourth, the research sample consists of individuals who were eligible for and completed 10 weeks of a medication trial. This subgroup may not fully represent the general population of patients with first-episode psychosis, as trial participants could differ in clinical characteristics, treatment adherence or other prognostic factors influencing prognosis. Consequently, this limited representativeness may have affected the prognostic accuracy of predictors.

Fifth, the use of binary (remission/no remission) outcome measures (PANSS and PSP) imposed a strict cut-off, meaning that patients with meaningful improvement just below the remission threshold were classified as non-remission, leading to information loss. While applied consistently, this may not fully reflect clinical improvement, as small but meaningful changes in PANSS or PSP scores are overlooked. Furthermore, this binary outcome definition limits the evaluation of predictions expressed as percentage chances of remission by PSY or MLM. Predictions may be deemed incorrect if a patient narrowly misses the cut-off or may appear correct if a patient barely meets the threshold. This can lead to both underestimation and overestimation of predictive performance.

Finally, the outcome measures were selected and rated by researchers and clinicians, rather than patients themselves.

### Recommendations for future research

Incorporating a broader range of prognostic factors may improve the predictive accuracy of both psychiatrists and MLMs. Machine learning techniques from fields such as weather forecasting,^27^ which successfully integrate complex data may offer strategies to improve psychiatric predictions. Future MLMs incorporating multimodal data such as neuroimaging, electroencephalogram, genetics and wearable data may enhance accuracy. Longitudinal data could also refine predictions.^17^ However, increasing the data collection burden might limit the model’s feasibility for routine clinical use.

Future research should also explore how direct clinical contact influences prognostic accuracy by comparing predictions based on case data with those made after in-person evaluations. If ‘clinical impression’ is found to predict outcomes, methods for integrating this into MLMs should be developed. One approach is to provide the MLM with audio and video recordings, enabling the model to derive information on speech patterns, posture, facial expressions and interactions with others. Another approach is to provide MLMs with descriptive diagnoses, which offer a more comprehensive and holistic understanding of the patient’s condition, aligning more closely with clinicians' impressions than standard disease classifications. In such a descriptive diagnosis, it would be important to also incorporate the perspective of patients and their informal caregivers, because previous research showed that the perspectives of patients, informal caregivers and healthcare professionals on barriers and facilitators of recovery can diverge and possibly complement each other.^28^

Larger sample sizes and more balanced case distributions will also be important for detecting subtle effects, particularly in outcomes like functional remission. Extending the follow-up period beyond 10 weeks is crucial to capture long-term recovery, especially for functional outcomes. For personalised treatment, future research should prioritise patient-relevant outcome measures.^28^ Finally, comparing the decision-making processes of psychiatrists and MLMs could shed light on how they can complement each other.

### Clinical implications

Despite the comparable predictive performance, replacing psychiatrists with MLMs in prognosis prediction tasks is unlikely, due to the clinical responsibility and liability associated with medical decisions. A more realistic approach would involve using MLM predictions as a second opinion, as we did in this study. In this scenario, psychiatrists would retain full autonomy over their final decision, integrating the MLM output as one piece of supplementary information. Since we do not know in which cases psychiatrists should rely on the MLM, a potential strategy could be to consistently average the predictions of the psychiatrist and the MLM. This approach leverages the strengths of multiple sources to mitigate individual biases and errors.^29^ In the current study, this would have resulted in a mean post-MLM accuracy of 0.54 for symptomatic remission (actual values pre-MLM 0.52, post-MLM 0.51, MLM 0.50) and 0.79 for functional remission (actual values pre-MLM 0.72, post-MLM 0.72, MLM 0.79).

For MLMs to add value in clinical practice, several improvements are necessary. First, increasing the accuracy of the MLM would enhance its utility in refining psychiatrists’ predictions. Second, identifying when psychiatrists should rely on the MLM is critical. This could be achieved by improving the model’s ability to estimate the certainty of its predictions or by identifying cases that are hard to predict for psychiatrists.

Third, explaining model decisions is crucial for enabling psychiatrists to make more informed judgments about when to trust the model’s output. Model interpretability plays a key role in this, as it helps clinicians understand how specific patient features influence the model’s predictions. Further future research on model explanation techniques is imperative to provide clinicians with clearer insights into the model’s decision-making process and improve their trust in artificial intelligence models.^30^

## Supplementary material

10.1136/bmjment-2025-301594 online supplemental file 1

## Data Availability

Data are available upon reasonable request.

## References

[1] Catalan A, Richter A, Salazar de Pablo G (2021). Proportion and predictors of remission and recovery in first-episode psychosis: Systematic review and meta-analysis. Eur Psychiatry.

[2] Percie du Sert O, Unrau J, Dama M (2025). Latent Trajectories of Positive, Negative Symptoms and Functioning in Early Intervention Services for First-Episode Psychosis: A 2-Year Follow-Up Study. Schizophr Bull.

[3] Gade K, Köhler J, Klein P (2013). Predictors of symptomatic remission in first-episode psychosis outpatients treated with quetiapine: a naturalistic study. Int J Psychiatry Clin Pract.

[4] Del Fabro L, Bondi E, Serio F (2023). Machine learning methods to predict outcomes of pharmacological treatment in psychosis. Transl Psychiatry.

[5] Leighton SP, Upthegrove R, Krishnadas R (2019). Development and validation of multivariable prediction models of remission, recovery, and quality of life outcomes in people with first episode psychosis: a machine learning approach. Lancet Digit Health.

[6] Dwyer DB, Falkai P, Koutsouleris N (2018). Machine Learning Approaches for Clinical Psychology and Psychiatry. Annu Rev Clin Psychol.

[7] Meehan AJ, Lewis SJ, Fazel S (2022). Clinical prediction models in psychiatry: a systematic review of two decades of progress and challenges. Mol Psychiatry.

[8] Schulz P, Berney P (2004). Clinicians’ predictions of patient response to psychotropic medications. Dialogues Clin Neurosci.

[9] Lennon MJ, Harmer C (2023). Machine learning prediction will be part of future treatment of depression. *Aust N Z J Psychiatry*.

[10] Van den Brink N, Holbrechts B, Brand PLP (2019). Role of intuitive knowledge in the diagnostic reasoning of hospital specialists: a focus group study. BMJ Open.

[11] Parnas J, Parnas AU (2024). Refining the Diagnostic Criteria for Schizophrenia: An Infinite Task. Schizophr Bull.

[12] Giel R, Wiersma D, de Jong PA (1984). Prognosis and outcome in a cohort of patients with non-affective functional psychosis. Eur Arch Psychiatry Neurol Sci.

[13] Atakan Z, Cliff G, Cooper JE (1990). Can psychiatrists predict the one-year outcome of schizophrenia?. Soc Psychiatry Psychiatr Epidemiol.

[14] Kahn RS, Winter van Rossum I, Leucht S (2018). Amisulpride and olanzapine followed by open-label treatment with clozapine in first-episode schizophrenia and schizophreniform disorder (OPTiMiSE): a three-phase switching study. Lancet Psychiatry.

[15] Andreasen NC, Carpenter WT, Kane JM (2005). Remission in schizophrenia: proposed criteria and rationale for consensus. Am J Psychiatry.

[16] Morosini PL, Magliano L, Brambilla L (2000). Development, reliability and acceptability of a new version of the DSM-IV Social and Occupational Functioning Assessment Scale (SOFAS) to assess routine social functioning. Acta Psychiatr Scand.

[17] van Opstal DPJ, Kia SM, Jakob L (2025). Psychosis Prognosis Predictor: A continuous and uncertainty-aware prediction of treatment outcome in first-episode psychosis. Acta Psychiatr Scand.

[18] Castor EDC (2019). Castor electronic data capture 2019. https://castoredc.com.

[19] Koo TK, Li MY (2016). A Guideline of Selecting and Reporting Intraclass Correlation Coefficients for Reliability Research. J Chiropr Med.

[20] Spengler PM, Pilipis LA (2015). A comprehensive meta-reanalysis of the robustness of the experience-accuracy effect in clinical judgment. J Couns Psychol.

[21] Peralta V, García de Jalón E, Moreno-Izco L (2022). Long-Term Outcomes of First-Admission Psychosis: A Naturalistic 21-Year Follow-Up Study of Symptomatic, Functional and Personal Recovery and Their Baseline Predictors. Schizophr Bull.

[22] Vila-Badia R, Butjosa A, Del Cacho N (2021). Types, prevalence and gender differences of childhood trauma in first-episode psychosis. What is the evidence that childhood trauma is related to symptoms and functional outcomes in first episode psychosis? A systematic review. Schizophr Res.

[23] Le TP, Ventura J, Subotnik KL (2024). Intrinsic motivation predicts cognitive and functional gains during coordinated specialty care for first-episode schizophrenia. Schizophr Res.

[24] Taub S, Krivoy A, Whiskey E (2022). New approaches to antipsychotic medication adherence - safety, tolerability and acceptability. Expert Opin Drug Saf.

[25] van Dee V, Schnack HG, Cahn W (2023). Systematic review and meta-analysis on predictors of prognosis in patients with schizophrenia spectrum disorders: An overview of current evidence and a call for prospective research and open access to datasets. Schizophr Res.

[26] Santesteban-Echarri O, Paino M, Rice S (2017). Predictors of functional recovery in first-episode psychosis: A systematic review and meta-analysis of longitudinal studies. Clin Psychol Rev.

[27] Topol EJ (2024). Medical forecasting. Science.

[28] van Dee V, Swildens W, Schnack HG (2025). In Pursuit of Recovery: A Comparative Study of Stakeholder Perspectives on Outcomes of People with Psychosis. Community Ment Health J.

[29] Kattan MW, O’Rourke C, Yu C (2016). The Wisdom of Crowds of Doctors: Their Average Predictions Outperform Their Individual Ones. Med Decis Making.

[30] van Dee V, Kia SM, Winter-van Rossum I (2023). Revealing the impact of psychiatric comorbidities on treatment outcome in early psychosis using counterfactual model explanation. Front Psychiatry.

