# Supplementary material for: Prognostic predictions in psychosis: exploring the complementary role of machine learning models
Source: BMJ Ment Health. 2025 Jun 26;28(1):e301594. doi: 10.1136/bmjment-2025-301594 (PMC12207152; doi:10.1136/bmjment-2025-301594)
Supplement: online supplemental file 1 [file bmjment-28-1-s001.pdf]

Prognostic Predictions in Psychosis: Exploring the Complementary Role of Machine Learning Models

Supplemental materials

Content

Supplemental material	Page(s)
Supplement 1 - Characteristics of the included 66 patients from the OPTiMiSE trial	2
Supplement 2 Example of Castor survey 'Psychiatrists versus machine in psychosis prognosis prediction'	3-6
Supplement 3 - Predictive performances of psychiatrists and MLM	7-10
Supplement 4 - Interrater agreement by intraclass correlation coefficients (ICCs)	11-12
Supplement 5 - Relationships and distributions of predictions by multidimensional scaling plots	13-14
Supplement 6 - Changes in predictions by psychiatrists post-MLM	15
Supplement 7 - Visualisation of accuracy of predictions pre- and post-MLM	16-17
Supplement 8 - Relative similarity between cases based on patient characteristics	18-19

Supplement 1 - Characteristics of the included 66 patients from the OPTiMiSE trial

Baseline	
Sex, male	79%
Mean age, years (SD)	25.3 (0.8)
DSM-classification	
- schizophrenia	67%
- schizophreniform disorder	32%
- schizo-affective disorder	2%
Inpatient status	55%
Mean duration of current psychotic episode, months (SD)	2.5 (0.5)
(Volunteer)work or school	32%
Psychiatric comorbidities	30%
Symptomatic remission according to RSWG-criteria*	0%
Functional remission defined as PSP >70	12%
Outcome at week 10	
Symptomatic remission according to RSWG-criteria*	44%
Functional remission defined as PSP >70	12%

*only the symptom severity component of the RSWG criteria was used, not the time-component.

Supplement 2. Example of Castor survey 'Psychiatrists versus machine in psychosis prognosis prediction'

Introduction

The following survey contains sensitive information about patients. By clicking the 'Next' button, the participant agrees not to disclose any information about the questionnaire to third parties and to close all forms containing sensitive information after completing them.

General respondent information

Question	Answer
Age	
Sex	○ Female<br>○ Male<br>○ I don't want to answer
Country of birth	○ Afghanistan<br>○ Albania<br>○ Algeria<br>○ Andorra<br>○ Angola<br>○ Antigua and Barbuda<br>○ Argentina<br>➔ Etc.

Case 1 Patient information

Please use control+click (windows) or command+click (mac) **[here]** to find the information about this patient in a new window.

After opening the new patient's browser tab, you can move on to the prognosis questions by clicking the 'next' button on the current page.

WARNING: To avoid confusion, please make sure you've closed previous tabs with patient information before opening this new one.

Case 1 – Prediction pre-MLM

WARNING: Next, you will be asked to provide your estimated prognosis for this patient. Then the estimated prognosis of the machine learning model will be presented to you and, based on this information, you get the opportunity to change your estimated prognosis.

Question	Answer
What do you think is the probability to achieve SYMPTOMATIC remission (RSWG) after 10 weeks?	no remission ------------------------------------ remission<br>(0%) (100%)
Which element of the provided information were most important for your prediction?	[ ]

What do you think is the probability to achieve FUNCTIONAL remission (based on PSP questionnaire, score >70) after 10 weeks?	no remission ------------------------------------ remission<br>(0%) (100%)
Which element of the provided information were most important for your prediction?	

Case 1 – Prediction post-MLM

WARNING: it is not allowed to return to previous pages to edit answers! When analyzing the results, controls will be carried out to ensure that no subsequent changes have been made.

The machine learning model predicts 82% chance of symptomatic remission for this patient, and it is certain about this prediction.

Question	Answer
Your predictions of the probability of SYMPTOMATIC remission (RSWG) after 10 weeks:	75%
Would you like to change your prediction of the probability of SYMPTOMATIC remission?	o Yes<br>o No
If 'Would you like to change your prediction of SYMPTOMATIC remission?' is equal to 'Yes' – answer this question:<br>What do you think is the probability to achieve SYMPTOMATIC remission (RSWG) after 10 weeks?	no remission ------------------------------------ remission<br>(0%) (100%)

The machine learning model predicts 33% chance of functional remission for this patient, and it is certain about this prediction.

Question	Answer
Your predictions of the probability of FUNCTIONAL remission (PSP) after 10 weeks:	45%
Would you like to change your prediction of the probability of FUNCTIONAL remission?	o Yes<br>o No
If 'Would you like to change your prediction of FUNCTIONAL remission?' is equal to 'Yes' – answer this question:<br>What do you think is the probability to achieve FUNCTIONAL remission (PSP) after 10 weeks?	no remission ------------------------------------ remission<br>(0%) (100%)

Estimated predictive accuracy

Question	Answer

How do you estimate your own mean predictive accuracy in this study for symptomatic remission and for functional remission?<br><br>Symptomatic remission (range 0 – 100%)<br>Functional remission (range 0 – 100%)	 <br><br><br>… %<br>… %

Final information – Artificial Intelligence

Please answer the following questions with your opinion in mind regarding the prediction of patient prognosis by machine learning models (artificial intelligence) trained with large amounts of data.

Question	Answer
What is your level of trust in an artificial intelligence algorithm?	o Very high<br>o High<br>o Medium<br>o Low<br>o Very low
Did you often chance your prediction(s) after knowing the prediction of the machine learning model?	o Yes<br>o No
If 'Did you often chance your prediction(s) after knowing the prediction of the machine learning model?' is equal to 'Yes' – answer this question:<br><br>If Yes, why?	
If 'Did you often chance your prediction(s) after knowing the prediction of the machine learning model?' is equal to 'No' – answer this question:<br><br>If No, why?	

Final information – Ecological value

Did the provided patient information correspond to the information you collect during your clinical work? (except the face to face contact)	o Yes<br>o No
If 'Did the provided patient information correspond to the information you collect during your clinical work?' is equal to 'No' – answer this question:<br><br>What information did you miss or did you not use?	

Final information – Fatigue

How tired are you at this moment?	o Not at all<br>o Somewhat<br>o Moderately<br>o Very much
Do you think your level of fatigue may have affected the quality of your predictions?	o Yes<br>o No

Final information – End of questionnaire

Thank you for participating in our survey!

Supplement 3 - Predictive performances of psychiatrists and MLM

Participants are labeled by group number (G1-4) and psychiatrist (P) or resident (R) status

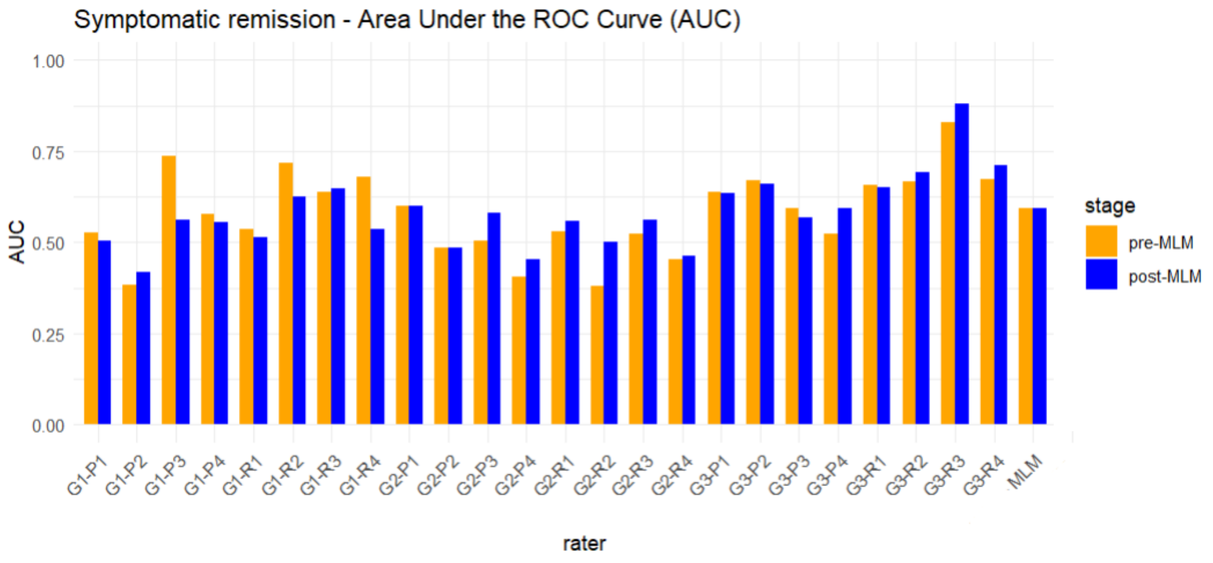

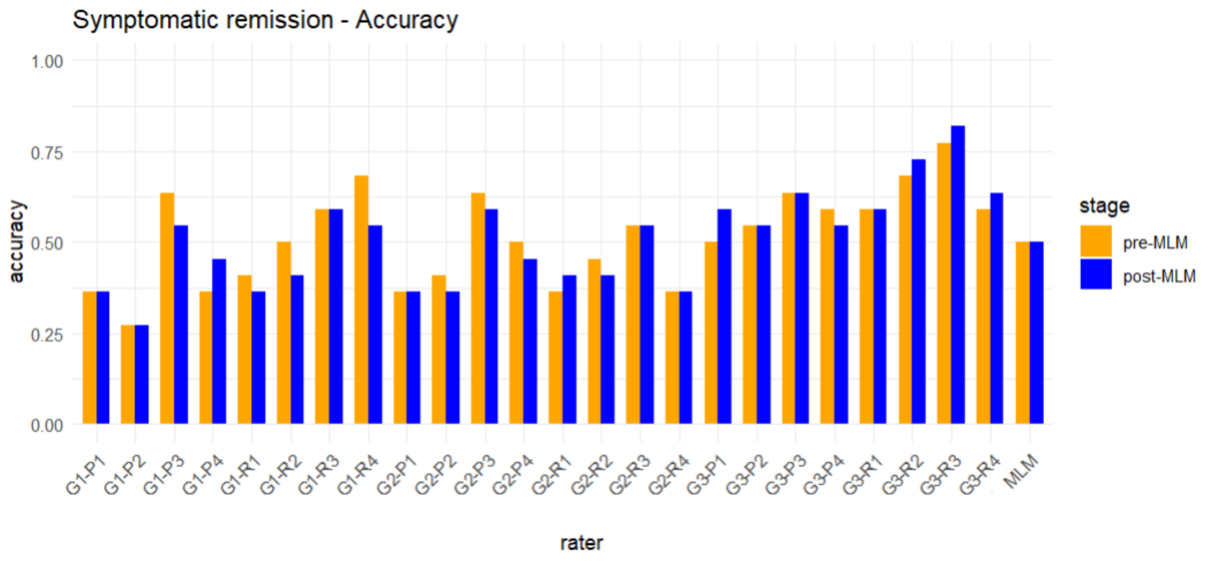

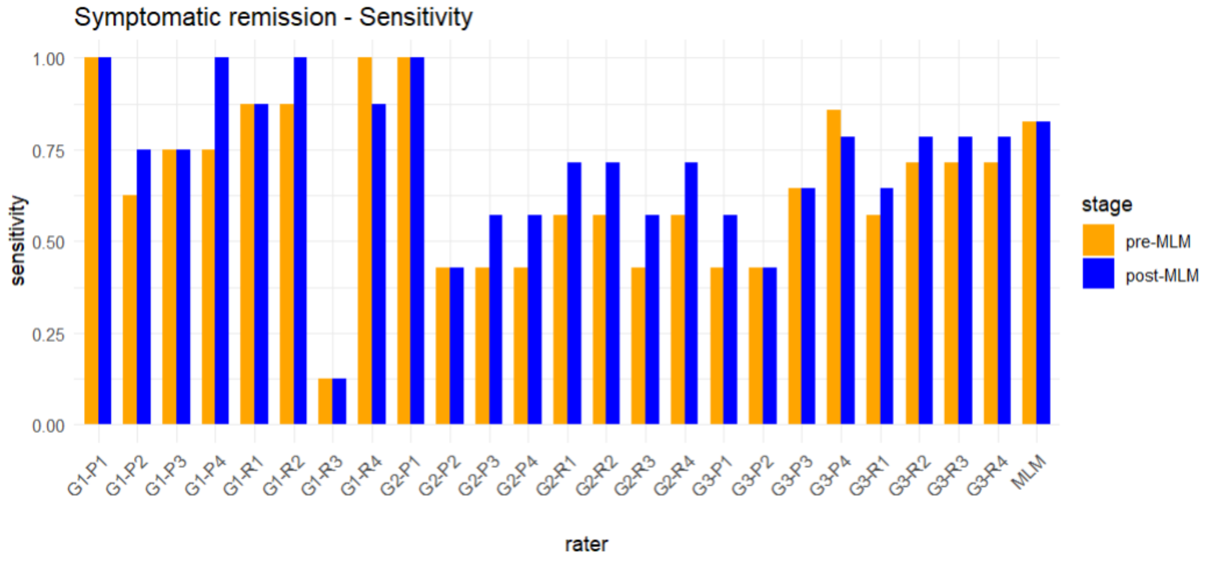

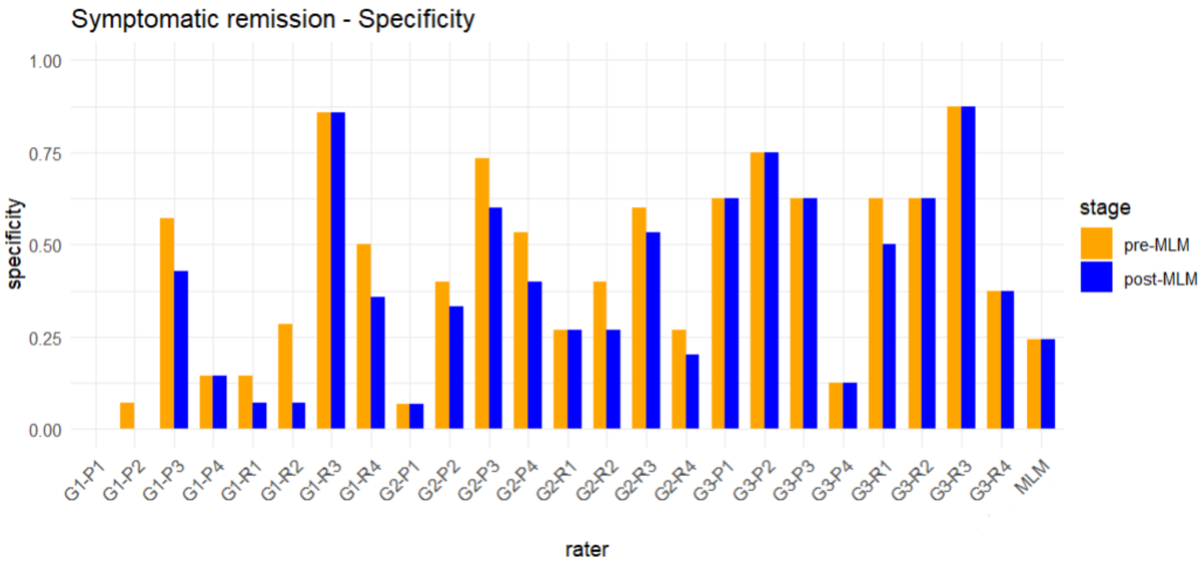
Symptomatic remission - Specificity
specificity
1.00
0.75
0.50
0.25
0.00
G1-P1 G1-P2 G1-P3 G1-P4 G1-R1 G1-R2 G1-R3 G1-R4 G2-P1 G2-P2 G2-P3 G2-P4 G2-R1 G2-R2 G2-R3 G2-R4 G3-P1 G3-P2 G3-P3 G3-P4 G3-R1 G3-R2 G3-R3 G3-R4 MLM
rater
stage
pre-MLM
post-MLM

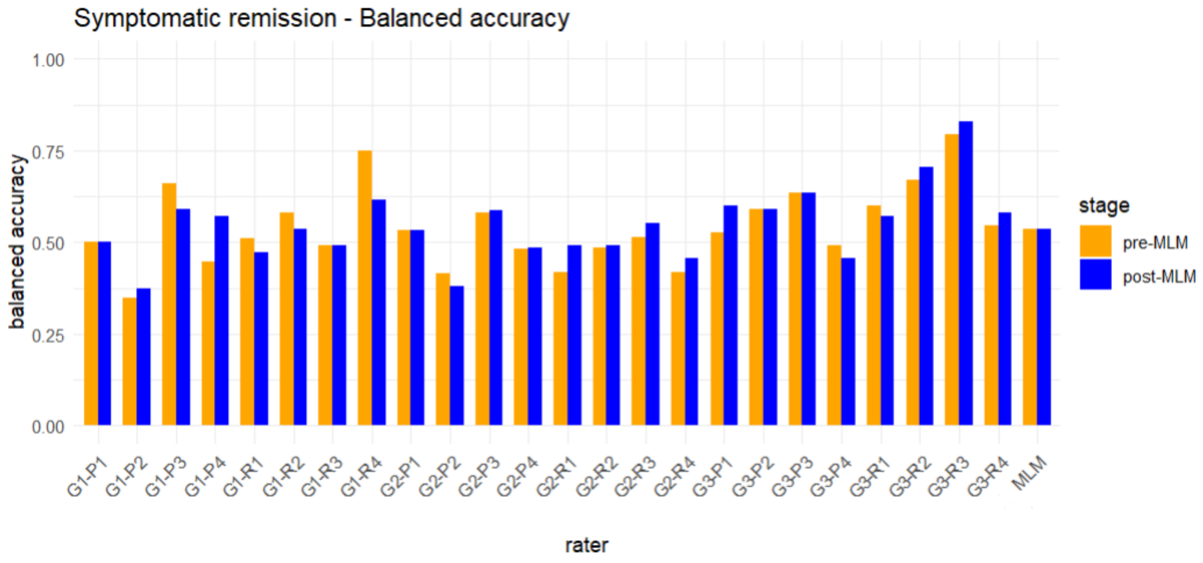
Symptomatic remission - Balanced accuracy
balanced accuracy
1.00
0.75
0.50
0.25
0.00
G1-P1 G1-P2 G1-P3 G1-P4 G1-R1 G1-R2 G1-R3 G1-R4 G2-P1 G2-P2 G2-P3 G2-P4 G2-R1 G2-R2 G2-R3 G2-R4 G3-P1 G3-P2 G3-P3 G3-P4 G3-R1 G3-R2 G3-R3 G3-R4 MLM
rater
stage
pre-MLM
post-MLM

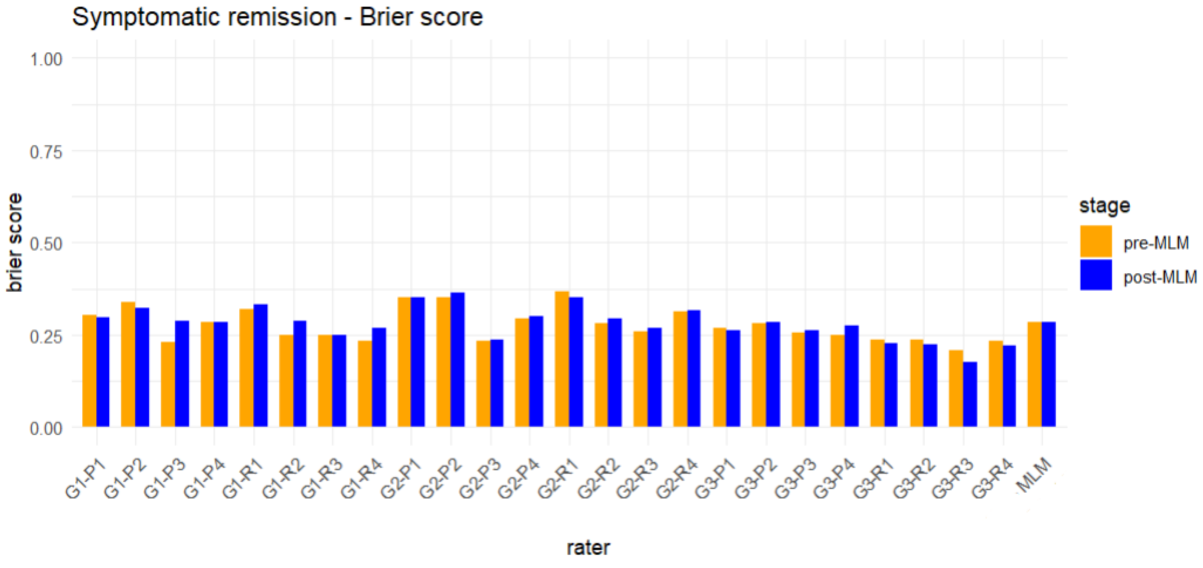
Symptomatic remission - Brier score
brier score
1.00
0.75
0.50
0.25
0.00
G1-P1 G1-P2 G1-P3 G1-P4 G1-R1 G1-R2 G1-R3 G1-R4 G2-P1 G2-P2 G2-P3 G2-P4 G2-R1 G2-R2 G2-R3 G2-R4 G3-P1 G3-P2 G3-P3 G3-P4 G3-R1 G3-R2 G3-R3 G3-R4 MLM
rater
stage
pre-MLM
post-MLM

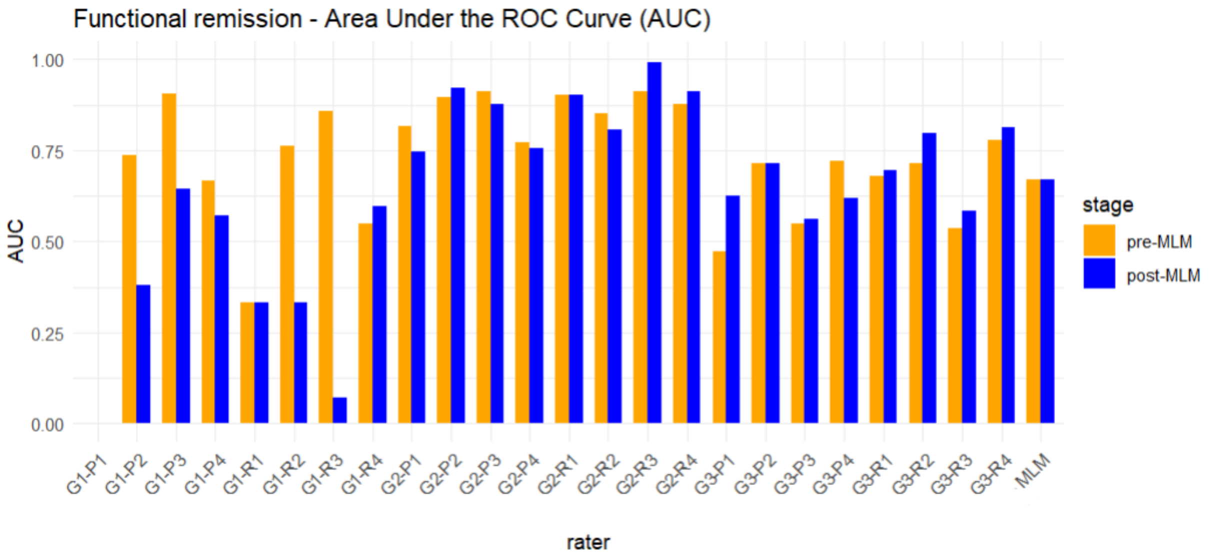
Functional remission - Area Under the ROC Curve (AUC)
AUC
1.00
0.75
0.50
0.25
0.00
G1-P1 G1-P2 G1-P3 G1-P4 G1-R1 G1-R2 G1-R3 G1-R4 G2-P1 G2-P2 G2-P3 G2-P4 G2-R1 G2-R2 G2-R3 G2-R4 G3-P1 G3-P2 G3-P3 G3-P4 G3-R1 G3-R2 G3-R3 G3-R4 MLM
rater
stage
pre-MLM
post-MLM

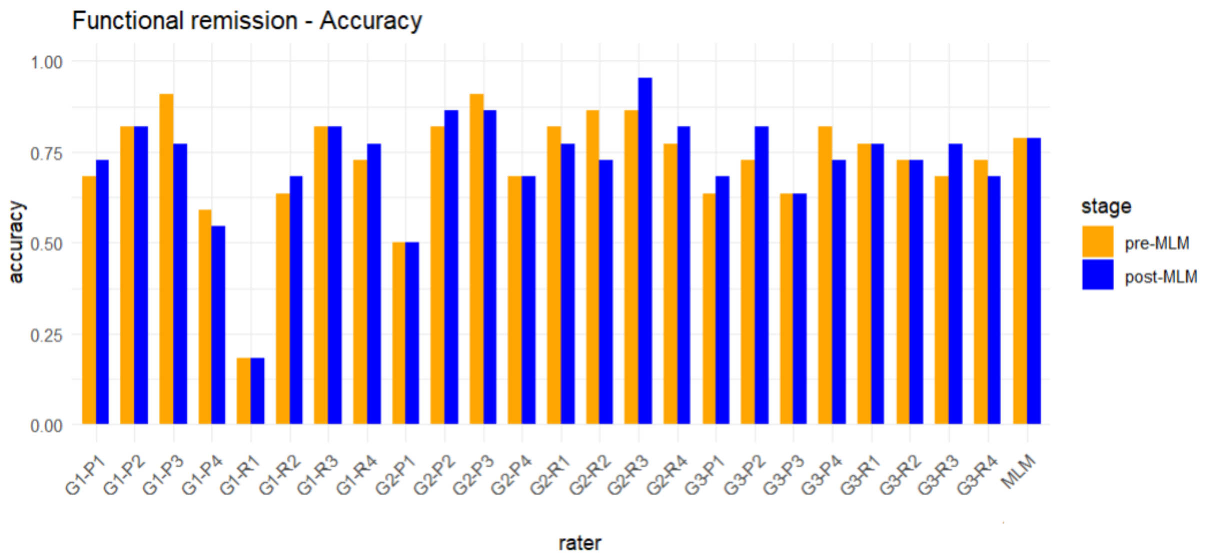
Functional remission - Accuracy
accuracy
1.00
0.75
0.50
0.25
0.00
G1-P1 G1-P2 G1-P3 G1-P4 G1-R1 G1-R2 G1-R3 G1-R4 G2-P1 G2-P2 G2-P3 G2-P4 G2-R1 G2-R2 G2-R3 G2-R4 G3-P1 G3-P2 G3-P3 G3-P4 G3-R1 G3-R2 G3-R3 G3-R4 MLM
rater
stage
pre-MLM
post-MLM

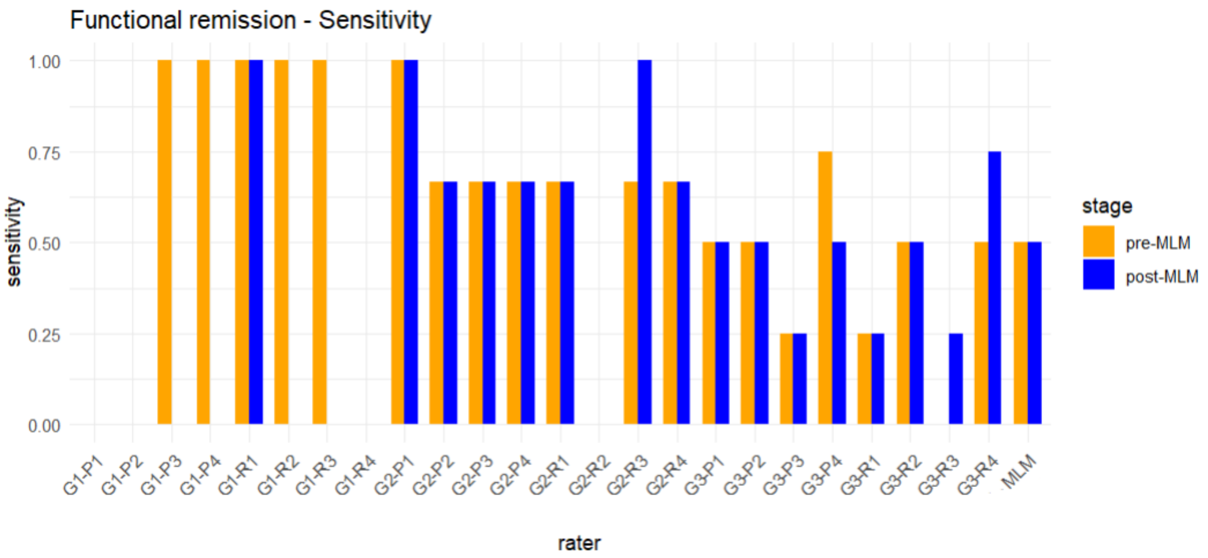
Functional remission - Sensitivity
sensitivity
1.00
0.75
0.50
0.25
0.00
G1-P1 G1-P2 G1-P3 G1-P4 G1-R1 G1-R2 G1-R3 G1-R4 G2-P1 G2-P2 G2-P3 G2-P4 G2-R1 G2-R2 G2-R3 G2-R4 G3-P1 G3-P2 G3-P3 G3-P4 G3-R1 G3-R2 G3-R3 G3-R4 MLM
rater
stage
pre-MLM
post-MLM

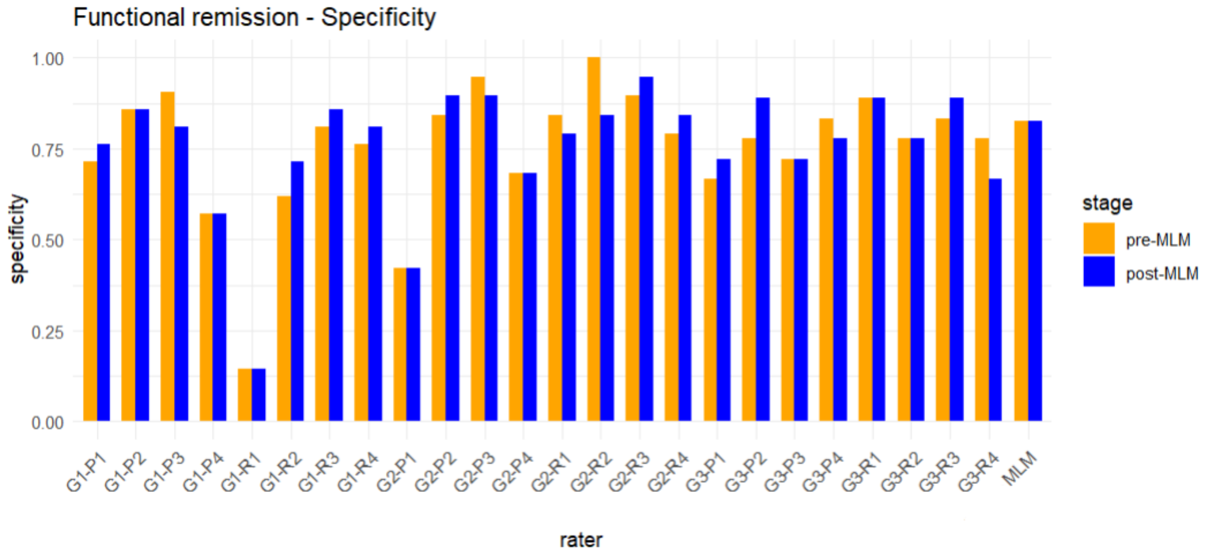
Functional remission - Specificity
specificity
1.00
0.75
0.50
0.25
0.00
G1-P1 G1-P2 G1-P3 G1-P4 G1-R1 G1-R2 G1-R3 G1-R4 G2-P1 G2-P2 G2-P3 G2-P4 G2-R1 G2-R2 G2-R3 G2-R4 G3-P1 G3-P2 G3-P3 G3-P4 G3-R1 G3-R2 G3-R3 G3-R4 MLM
rater
stage
pre-MLM
post-MLM

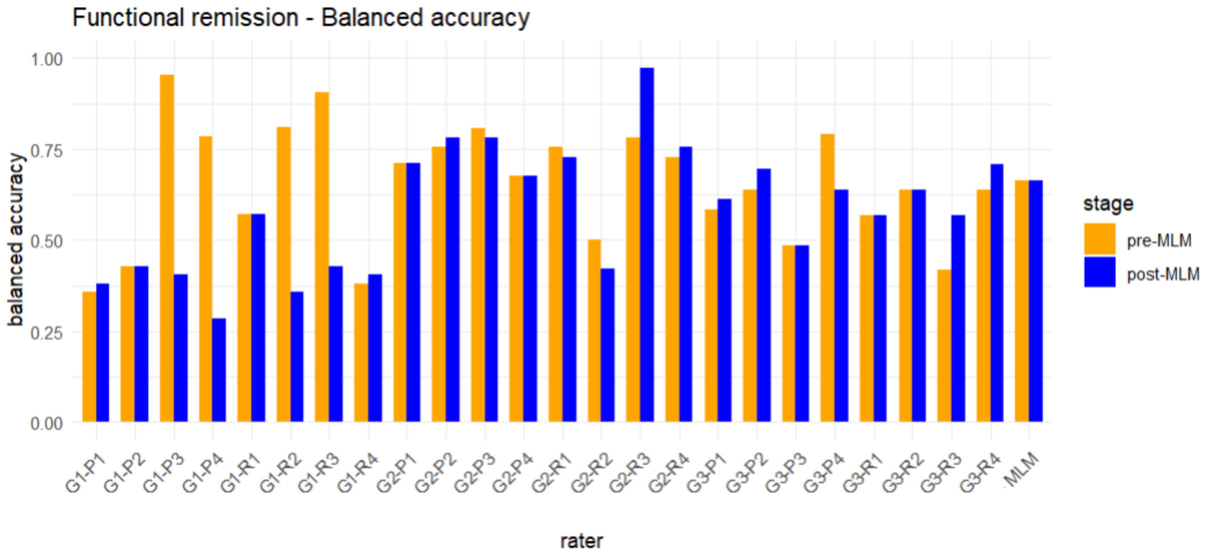
Functional remission - Balanced accuracy
balanced accuracy
1.00
0.75
0.50
0.25
0.00
G1-P1 G1-P2 G1-P3 G1-P4 G1-R1 G1-R2 G1-R3 G1-R4 G2-P1 G2-P2 G2-P3 G2-P4 G2-R1 G2-R2 G2-R3 G2-R4 G3-P1 G3-P2 G3-P3 G3-P4 G3-R1 G3-R2 G3-R3 G3-R4 MLM
rater
stage
pre-MLM
post-MLM

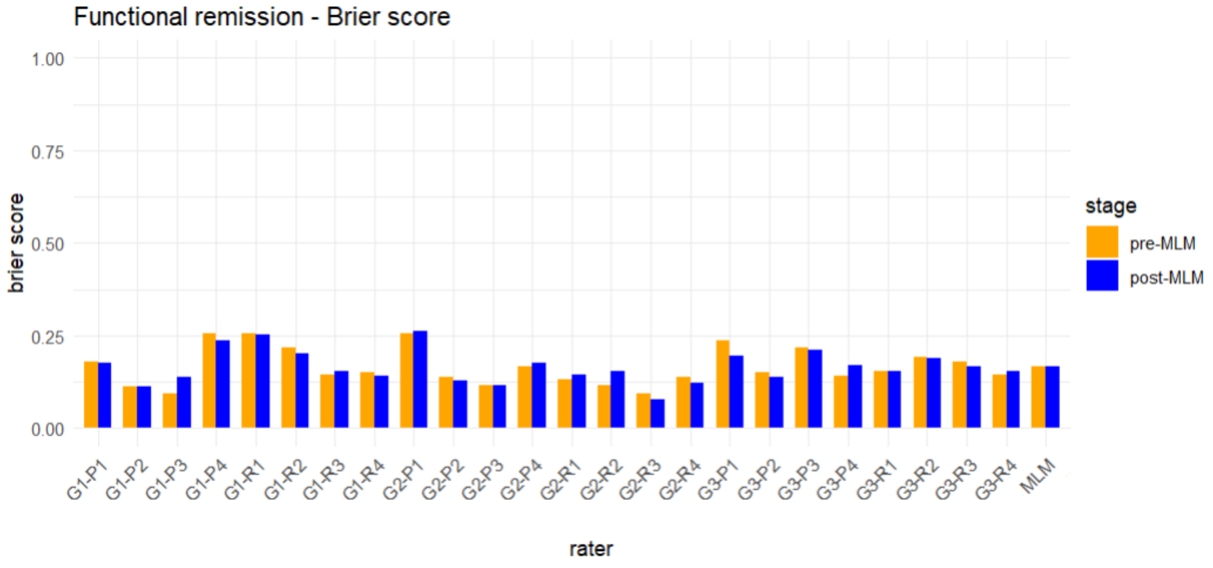
Functional remission - Brier score
brier score
1.00
0.75
0.50
0.25
0.00
G1-P1 G1-P2 G1-P3 G1-P4 G1-R1 G1-R2 G1-R3 G1-R4 G2-P1 G2-P2 G2-P3 G2-P4 G2-R1 G2-R2 G2-R3 G2-R4 G3-P1 G3-P2 G3-P3 G3-P4 G3-R1 G3-R2 G3-R3 G3-R4 MLM
rater
stage
pre-MLM
post-MLM

Supplement 4 - Interrater agreement by intraclass correlation coefficients (ICCs)
Participants are labeled by group number and psychiatrist or resident status

Symptomatic remission

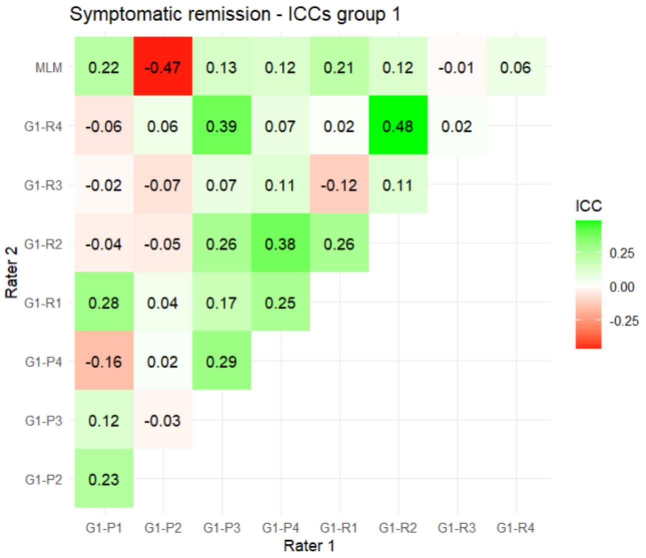

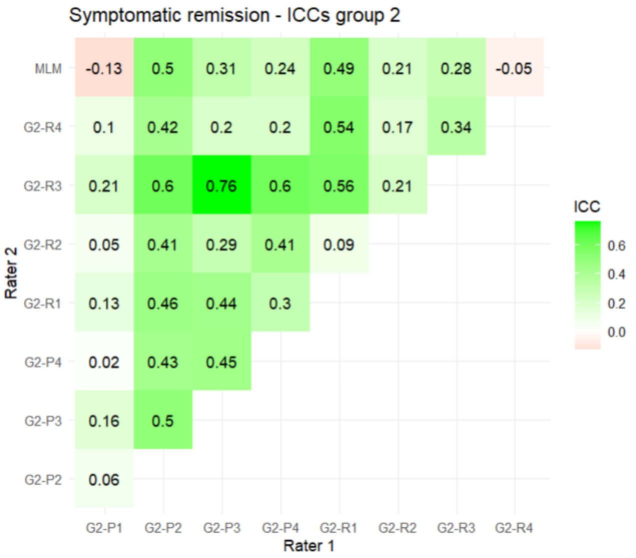

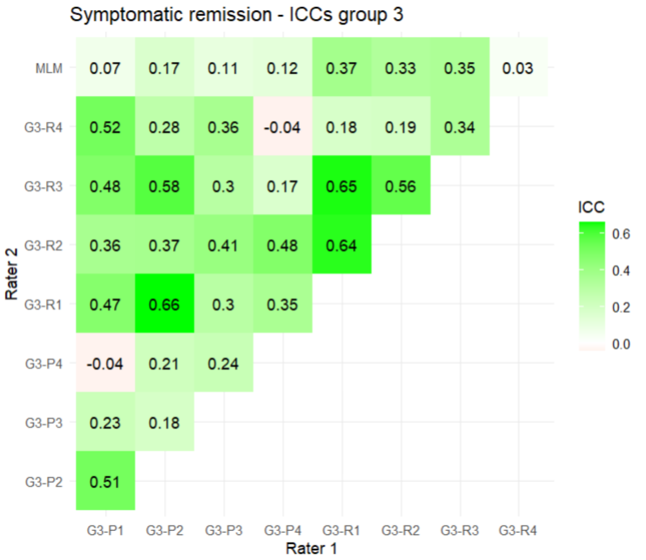

Functional remission

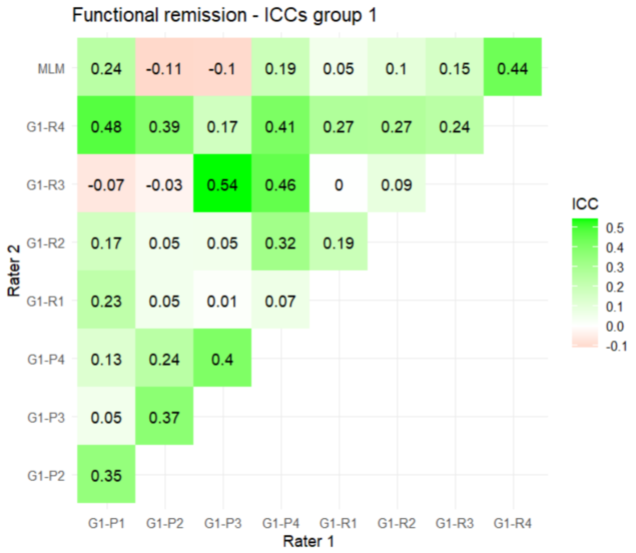

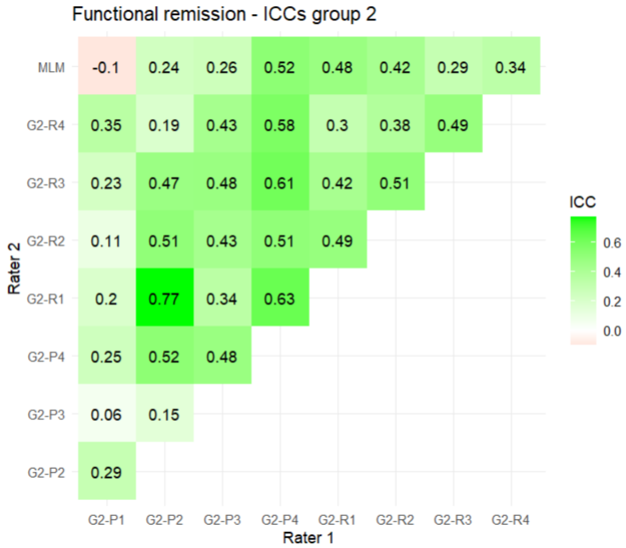

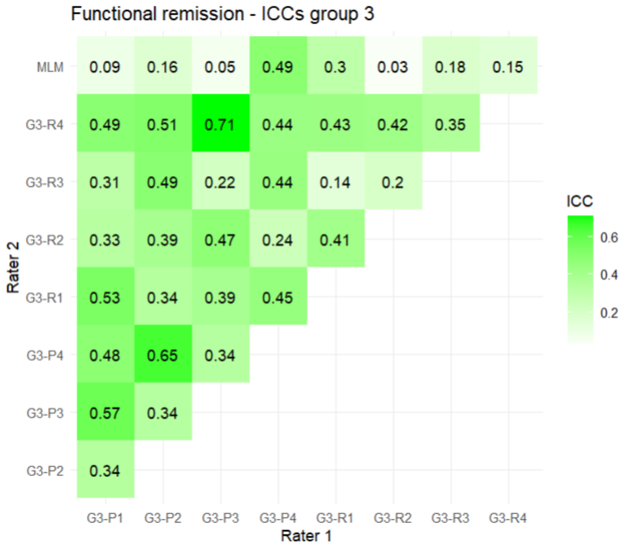

Supplement 5 - Relationships and distributions of predictions by multidimensional scaling plots

PSY pre-MLM PSY post-MLM MLM

Each psychiatrist is represented by a different color.

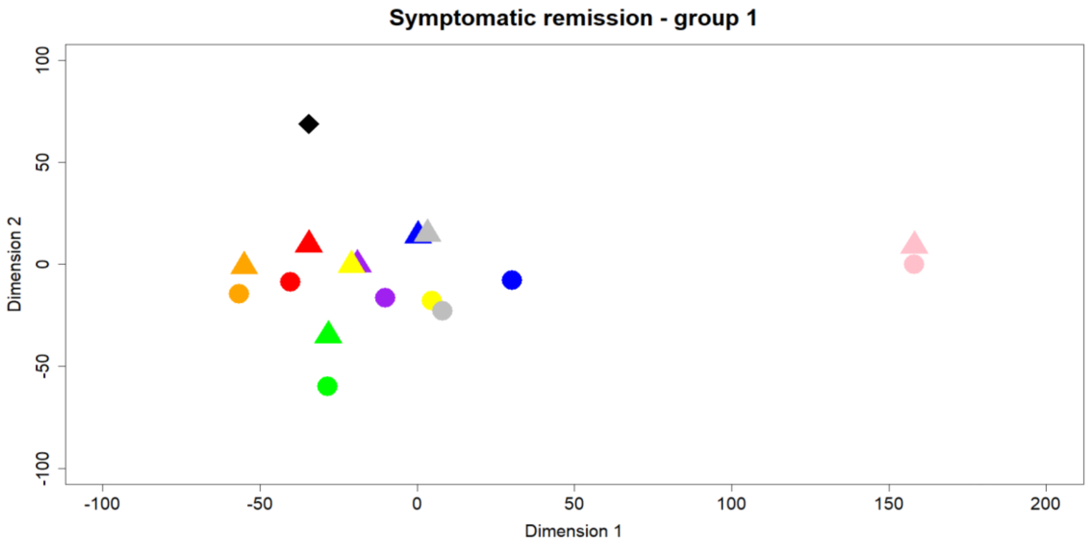

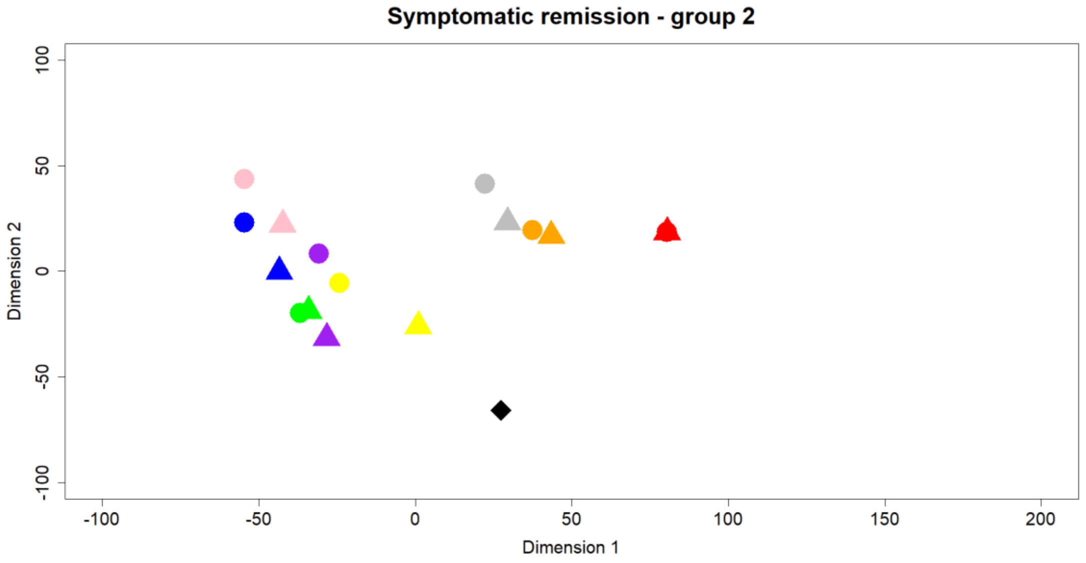

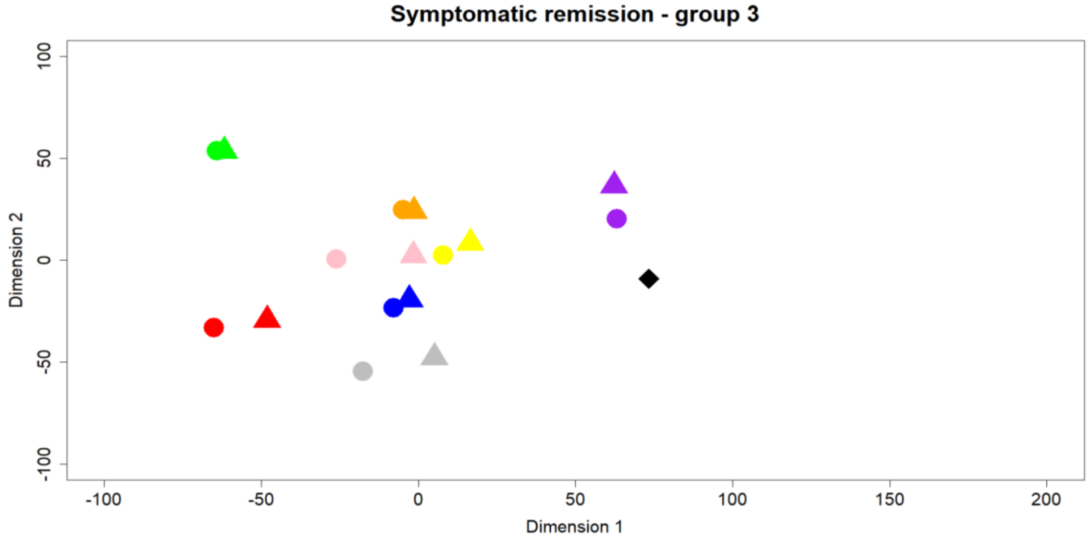

Functional remission - group 1

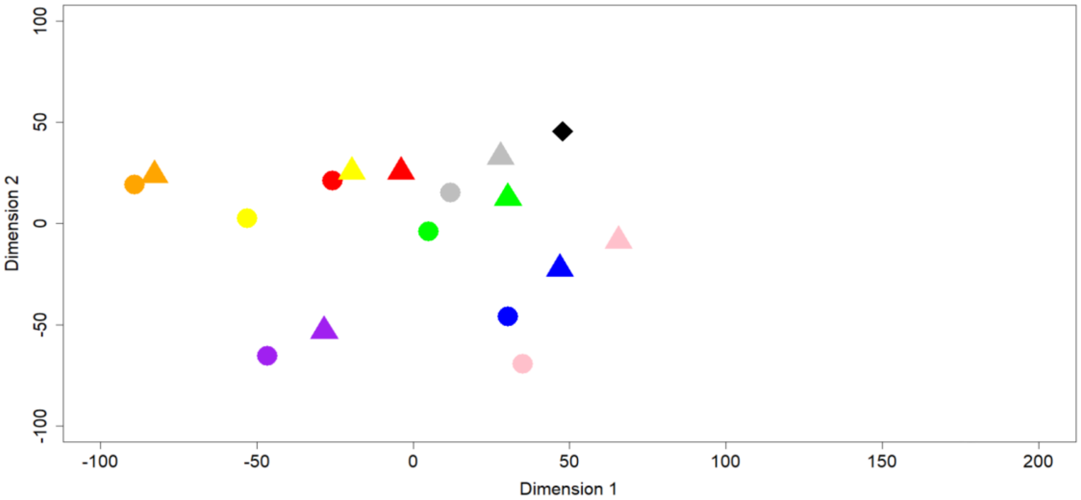

Functional remission - group 2

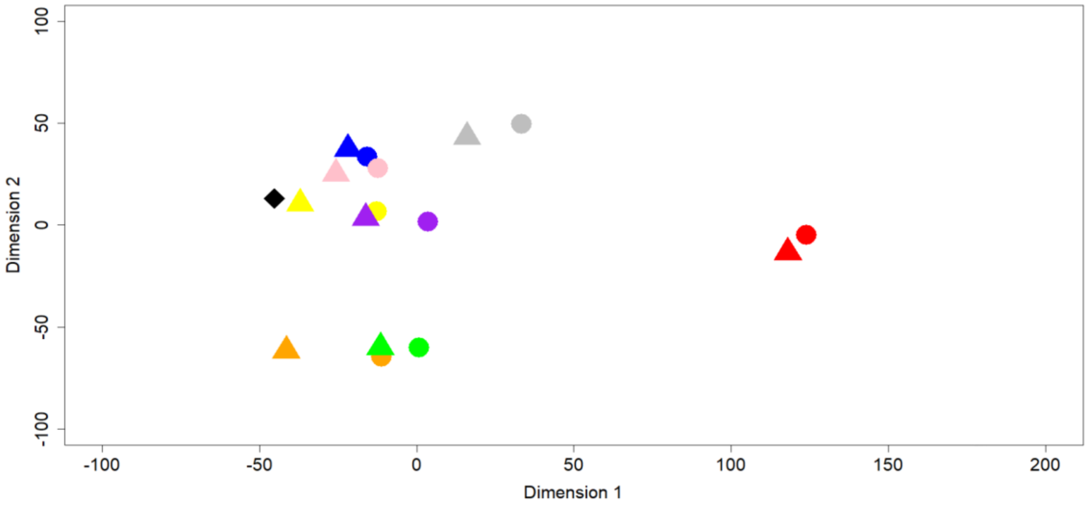

Functional remission - group 3

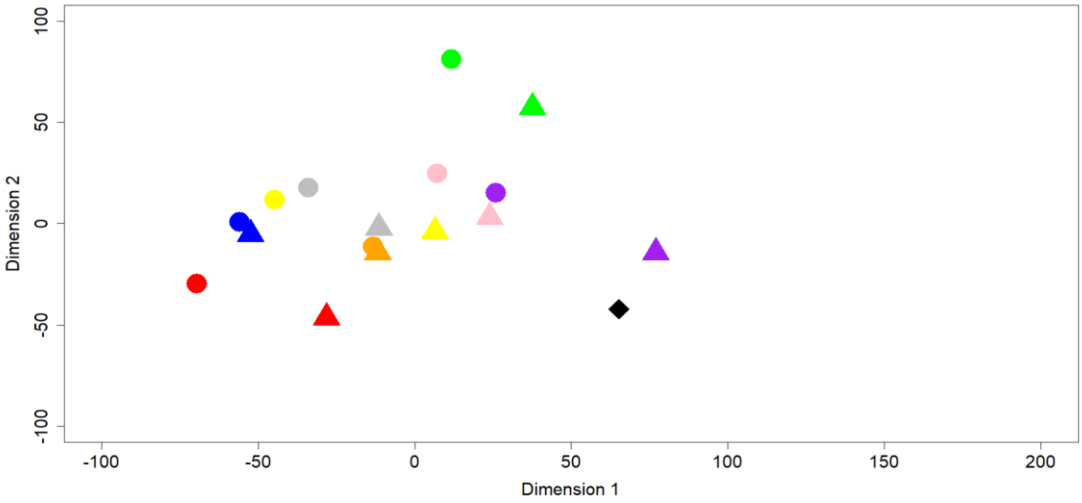

Supplement 6 - Changes in predictions by psychiatrists post-MLM

	Symptomatic remission[1]	Functional remission[2]
Change prediction post-MLM – *absolute*	25.6% (122/476)	26.3% (127/482)
Mean proportion of changed predictions	25.8% (range 0% to 77%)	26.7% (range 5% to 77%)
Mean abolute % of change in prognosis prediction	16.3% (range 0% to 28.5%)	21.3% (range 6% to 47%).
Change in correct direction	11.6% (55/476)	15.6% (75/482)
Change in wrong direction	14.1% (67/476)	10.8% (52/482)
Cases with at least one change	75.8% (50/66)	72.7% (48/66)
Change prediction post-MLM– *dichotomized*	9.2% (44/476)	8.7% (42/482)
Change in correct direction	4.2% (20/476)	4.4% (21/482)
Change in wrong direction	5.0% (24/476)	4.4% (21/482)
Cases with at least one change	39.4% (26/66)	37.9% (25/66)

[1] For 52 predictions spread over 11 cases there was no ML-prediction available for participants do to a technical error

[2] For 46 predictions spread over 9 cases there was no ML-prediction available for participants do to a technical error

Supplement 7 - Visualisation of accuracy of predictions pre- and post-MLM

SYMPTOMATIC REMISSION GROUP 1

	P1	P2	P3	P4	R1	R2	R3	R4
4	1	5	5	6	1	1	5	1
6	5	5	1	5	5	5	1	5
8	4	4	4	4	4	4	2	3
9	4	4	4	4	4	4	2	2
17	4	4	4	2	4	4	2	4
20	1	1	1	1	1	1	5	1
21	1	1	6	1	1	1	5	1
26	4	4	4	4	4	4	2	2
29	1	1	1	1	1	1	5	1
31	1	6	1	1	1	1	5	1
35	4	4	4	4	4	4	2	4
38	1	5	1	6	1	6	5	1
39	4	3	4	4	4	4	4	4
41	4	4	3	4	4	4	2	4
44	4	4	2	4	4	3	2	4
52	4	4	2	4	4	3	2	2
53	9	9	2	4	2	8	8	4
54	2	2	2	2	2	2	4	3
57	4	4	3	4	3	3	4	3
58	5	5	1	1	5	5	1	1
60	9	9	1	5	5	9	8	1
64	8	8	3	2	4	8	8	2

SYMPTOMATIC REMISSION GROUP 2

	P1	P2	P3	P4	R1	R2	R3	R4
1	1	1	1	1	1	1	1	1
3	4	2	2	3	4	2	2	2
7	4	4	3	4	2	4	3	3
11	1	1	1	1	1	1	1	1
15	1	1	1	1	1	1	1	1
19	4	3	4	4	4	3	4	2
23	4	4	4	4	4	4	4	4
25	4	4	4	4	4	4	4	4
28	1	5	5	6	1	5	5	1
30	4	4	4	4	4	4	4	2
34	1	5	5	5	5	5	5	5
36	2	4	3	3	2	4	2	4
40	4	2	8	2	9	9	8	9
42	5	1	8	1	8	8	8	9
43	1	5	6	5	6	6	6	6
46	4	4	2	2	4	2	2	4
55	4	4	2	4	4	4	4	4
59	5	1	1	1	5	1	1	5
61	5	1	8	5	9	8	9	9
62	4	4	2	2	4	4	2	4
63	5	5	8	1	8	9	8	9
65	1	5	5	5	5	1	5	5

SYMPTOMATIC REMISSION GROUP 3

	P1	P2	P3	P4	R1	R2	R3	R4
2	1	1	1	1	1	1	1	1
5	2	2	2	2	2	2	2	4
10	5	5	1	1	5	5	5	5
12	8	2	2	2	8	8	8	8
13	1	5	1	6	1	6	1	1
14	4	4	2	4	4	2	2	2
16	6	5	5	1	1	1	1	1
18	4	4	4	4	4	2	2	4
22	5	1	1	1	1	1	1	6
24	5	5	5	5	5	5	1	5
27	1	1	1	1	1	1	1	1
32	2	2	2	4	3	4	2	2
33	4	4	3	3	7	2	4	3
37	4	2	4	4	4	4	4	4
45	1	1	5	5	1	1	1	5
47	6	1	6	1	1	1	1	1
48	1	1	1	1	1	1	1	1
49	2	2	2	4	2	2	2	4
50	8	4	4	3	9	8	8	8
51	8	1	1	5	8	9	8	8
56	5	5	5	1	5	1	6	6
66	9	5	1	1	9	9	9	8

1	man correct + ML correct -> man correct - nothing going on
2	man correct + ML wrong -> man correct - good that man did not listen to ML
3	man correct + ML wrong -> man wrong - man should not have listened to ML
4	man wrong + ML wrong -> man wrong - the hard cases
5	man wrong + ML correct -> man wrong - man should have listened to the ML
6	man wrong + ML correct -> man correct - ML helps man!
7	man wrong + ML wrong --> man correct -strange
8	man correct --> no correct ML data available for participant
9	man wrong --> no correct ML data available for participant

FUNCTIONAL REMISSION GROUP 1

	P1	P2	P3	P4	R1	R2	R3	R4
4	1	1	1	1	5	5	1	1
6	6	6	1	1	5	5	1	6
8	1	1	1	1	5	1	1	1
9	8	8	1	1	9	8	8	1
17	1	1	1	1	5	1	1	1
20	5	5	5	5	5	1	5	5
21	3	3	3	4	4	4	2	3
26	6	6	1	5	5	1	1	6
29	5	1	1	5	5	6	1	5
31	8	8	1	1	9	8	9	1
35	1	1	1	1	5	1	1	1
38	1	1	1	1	5	1	1	1
39	4	2	3	4	4	4	4	4
41	4	2	2	2	4	4	2	2
44	2	3	2	4	4	2	2	2
52	1	1	1	5	1	1	1	1
53	1	1	1	1	1	6	1	1
54	4	4	3	3	2	3	3	4
57	8	8	1	1	1	8	8	1
58	1	1	1	1	5	1	1	1
60	1	1	5	5	5	5	6	1
64	1	1	1	5	5	1	1	1

FUNCTIONAL REMISSION GROUP 2

	P1	P2	P3	P4	R1	R2	R3	R4
1	1	1	5	5	1	5	6	5
3	8	1	8	1	8	8	8	8
7	5	5	1	5	5	1	1	1
11	8	2	8	2	8	9	8	8
15	1	5	1	1	5	5	1	1
19	5	6	1	1	1	1	6	1
23	5	1	1	1	1	1	1	1
25	2	2	3	4	4	3	4	4
28	5	1	1	1	1	1	1	6
30	1	1	5	5	1	1	1	1
34	1	1	1	1	1	1	1	1
36	2	2	2	4	3	3	2	2
40	1	1	1	1	1	1	1	1
42	1	1	1	1	1	1	1	1
43	1	1	1	1	1	1	1	1
46	5	1	1	1	1	1	1	1
55	4	4	2	4	4	3	2	2
59	5	1	1	1	1	1	1	5
61	5	1	1	5	1	1	1	5
62	5	1	8	1	8	8	8	8
63	5	1	1	1	1	1	1	1
65	5	1	8	1	8	8	8	8

FUNCTIONAL REMISSION GROUP 3

	P1	P2	P3	P4	R1	R2	R3	R4
2	5	6	5	5	1	1	5	5
5	1	1	1	1	1	1	1	1
10	1	1	5	1	1	1	1	1
12	2	2	2	3	2	2	4	2
13	5	1	5	1	1	1	1	5
14	5	1	1	1	1	1	6	1
16	1	5	5	1	5	1	6	6
18	2	2	2	4	2	4	2	4
22	2	4	2	3	2	3	2	3
24	8	1	1	1	8	8	8	8
27	1	5	5	1	1	1	6	5
32	1	1	1	1	1	6	1	1
33	1	6	1	1	1	5	1	1
37	4	2	4	4	4	4	3	3
45	1	1	1	1	1	1	1	1
47	5	1	5	1	5	5	5	5
48	6	1	1	1	5	1	1	1
49	1	1	1	1	1	1	1	1
50	5	1	1	1	1	1	1	1
51	1	1	1	1	1	1	1	1
56	8	1	1	1	8	8	8	8
66	4	4	4	4	4	4	4	2

1	man correct + ML correct -> man correct - nothing going on
2	man correct + ML wrong -> man correct - good that man did not listen to ML
3	man correct + ML wrong -> man wrong - man should not have listened to ML
4	man wrong + ML wrong -> man wrong - the hard cases
5	man wrong + ML correct -> man wrong - man should have listened to the ML
6	man wrong + ML correct -> man correct - ML helps man!
7	man wrong + ML wrong --> man correct -strange
8	man correct --> no correct ML data available for participant
9	man wrong --> no correct ML data available for participant

Supplement 8 - Relative similarity between cases based on patient characteristics

The following baseline patient information* was used:

<u>Characteristics:</u>

- *Demographic:* age (con), sex (m/f), occupation (y/n), highest level of education (cat), living alone (yes/no).
- *Diagnostic:* DSM-IV classification (Schizophrenia / Schizoaffective disorder / Schizophreniform disorder), current treatment setting (Inpatient / Outpatient / Day Care / Other)

<u>Measurements:</u>

- Calgary Depression Scale for Schizophrenia (CDSS): total score (con)
- Subjective well-being under Neuroleptic Treatment Scale (SWN-K): total score (con)
- Mini International Neuropsychiatric Interview (MINI): mood disorder (y/n), anxiety disorder (y/n), substance abuse/dependence (y/n)
- Positive And Negative Syndrome Scale (PANSS): positive (con), negative (con), general (con), total score (con)
- Personal and Social Performance Scale (PSP): domain A (con), domain B (con), domain C (con), domain D (con), total score (con)
- Clinical Global Impression scale (CGI-severity): total score (con)

*All input features were standardised for use in the t-sne plot

t-SNE plot Symptomatic remission – all groups

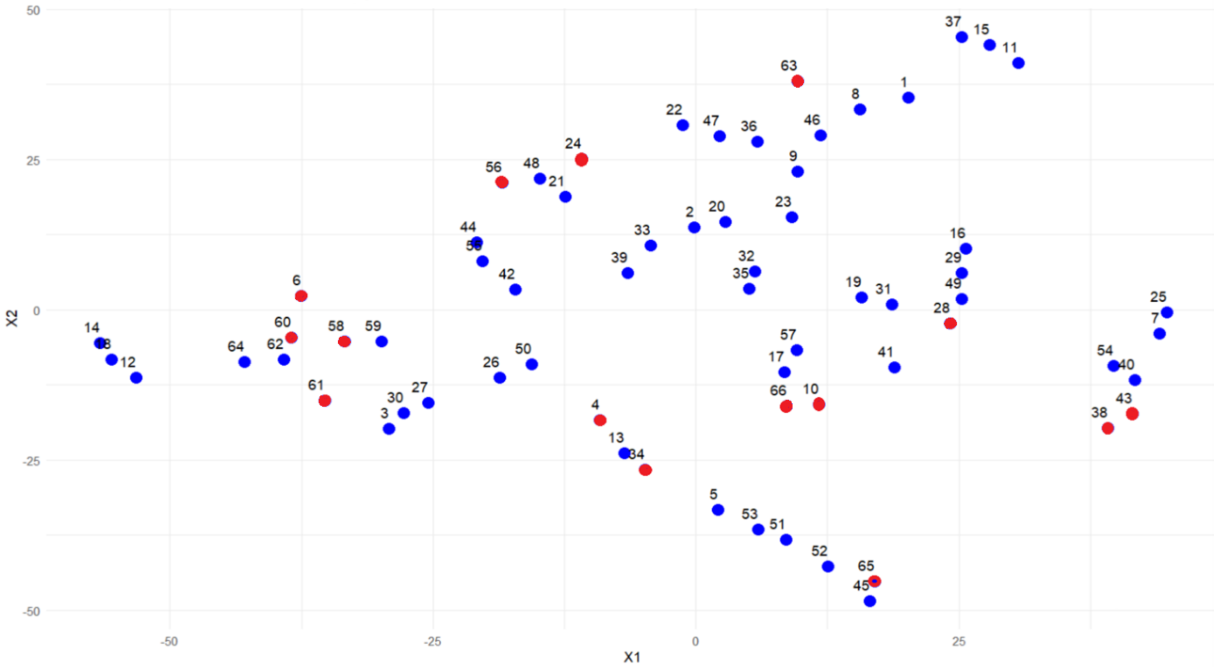

t-SNE Plot Functional remission – all groups

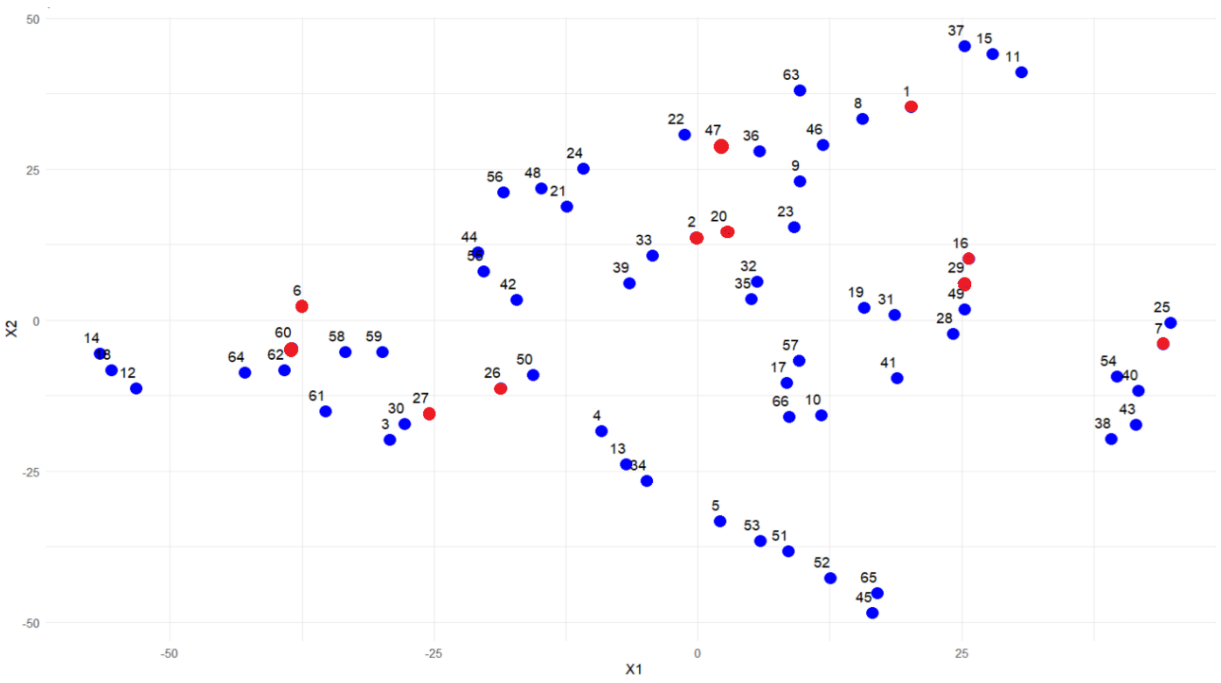

In the t-SNE plot, each case is represented by a data point. The position of the data points relative to each other is determined based on the case characteristics. Cases with more similarities are closer together than those that differ more. Red data points represent the hard cases; the cases that the MLM predicted correctly, while fewer than four psychiatrists did so correctly. The red points are not neatly grouped, meaning they cannot be identified based on case characteristics.
